# Supplementary material for: Paraxial Mesoderm Is the Major Source of Lymphatic Endothelium
Source: Dev Cell. 2019 Jul 22;50(2):247–255.e3. doi: 10.1016/j.devcel.2019.04.034 (PMC6658618; doi:10.1016/j.devcel.2019.04.034)
Supplement: Document S1. Figures S1–S4 [file mmc1.pdf]

Developmental Cell, Volume *50*

Supplemental Information

Paraxial Mesoderm Is the Major Source of Lymphatic Endothelium

Oliver A. Stone and Didier Y.R. Stainier

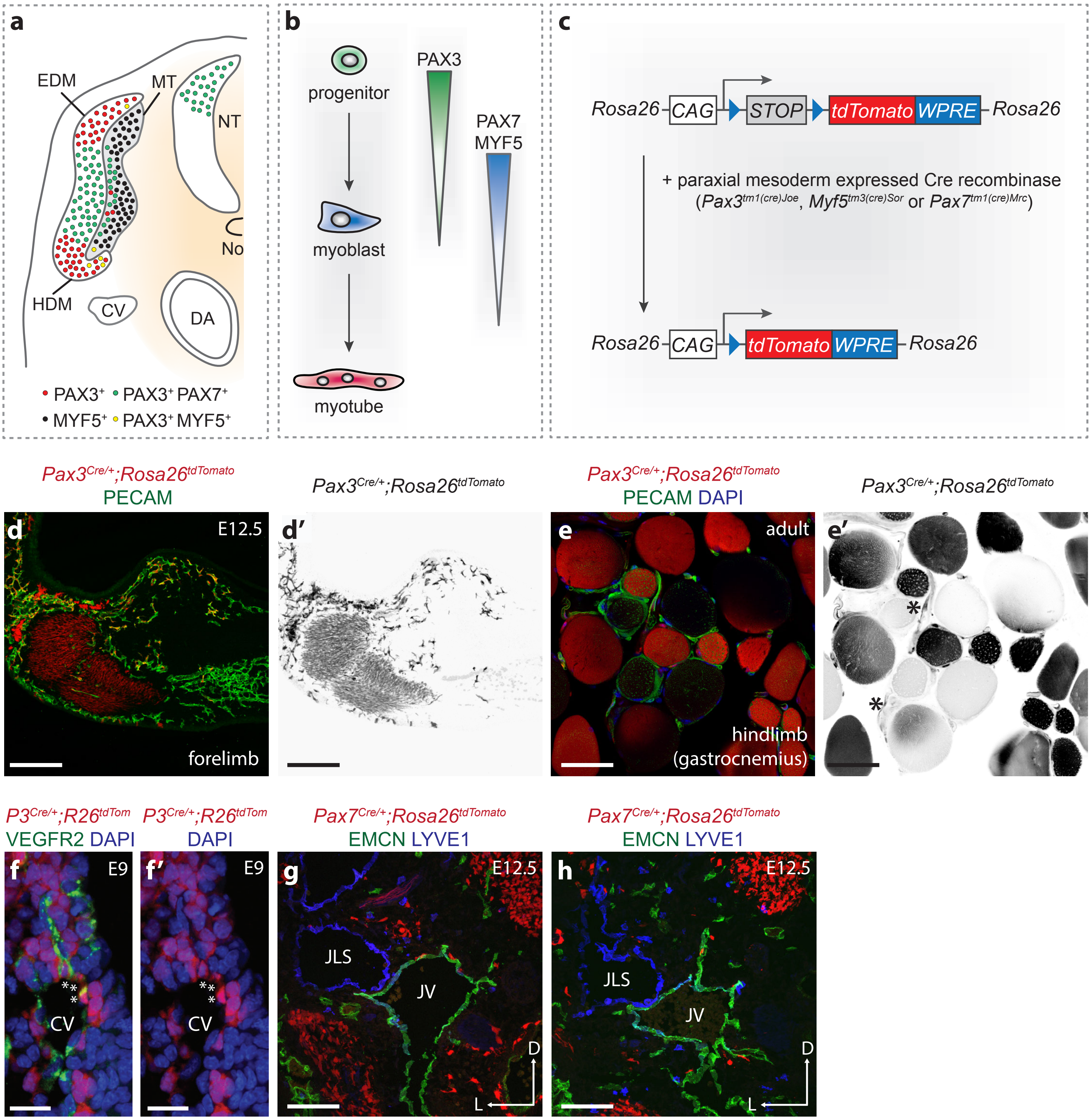
a
EDM
MT
NT
No
HDM
CV
DA
PAX3+ PAX3+ PAX7+
MYF5+ PAX3+ MYF5+
b
progenitor
PAX3
PAX7
MYF5
myoblast
myotube
c
Rosa26 CAG STOP tdTomato WPRE Rosa26
+ paraxial mesoderm expressed Cre recombinase
(Pax3tm1(cre)Joe, Myf5tm3(cre)Sor or Pax7tm1(cre)Mrc)
Rosa26 CAG tdTomato WPRE Rosa26
Pax3Cre/+;Rosa26tdTomato
PECAM
d
E12.5
forelimb
Pax3Cre/+;Rosa26tdTomato
d'
Pax3Cre/+;Rosa26tdTomato
PECAM DAPI
e
adult
hindlimb
(gastrocnemius)
Pax3Cre/+;Rosa26tdTomato
e'
P3Cre/+;R26tdTom
VEGFR2 DAPI
f
E9
CV
P3Cre/+;R26tdTom
DAPI
f'
E9
CV
Pax7Cre/+;Rosa26tdTomato
EMCN LYVE1
g
E12.5
JLS
JV
D
L
Pax7Cre/+;Rosa26tdTomato
EMCN LYVE1
h
E12.5
JLS
JV
D
L

Figure S1: Related to Figure 1. Analysis of paraxial mesodermal cell contribution to the developing vasculature.

Schematic representations of the embryonic expression of PAX3, PAX7 and MYF5 in the developing dermomyotome, myotome and neural tube (neural crest) (**a**), lineage commitment during embryonic myogenesis (**b**) and the strategy used to lineage trace cells from the paraxial mesoderm (**c**). Immunofluorescence for tdTomato and PECAM on cryosections of embryonic $Pax3^{Cre/+};Rosa26^{tdtomato}$ forelimb (**d**-**d**') and tdTomato, PECAM and DAPI on cryosections of adult $Pax3^{Cre/+};Rosa26^{tdtomato}$ gastrocnemius (**e**-**e**'). (**f**-**f**') Immunofluorescence for tdTomato and VEGFR2 on transverse cryosections from a $Pax3^{Cre/+};Rosa26^{tdtomato}$ embryo. (**g**, **h**) Immunofluorescence for tdTomato, EMCN and LYVE1 on transverse cryosections from $Pax7^{Cre/+};Rosa26^{tdtomato}$ embryos. (CV - cardinal vein; DA - dorsal aorta; HDM - hypaxial dermomyotome; EDM - epaxial dermomyotome; MT - myotome; No - notochord; NT - neural tube; JLS - jugular lymph sac; JV - jugular vein). Scale bars: **d, d'**, **g**, **h** – 100 µm; **e**-**f**' – 50 µm.

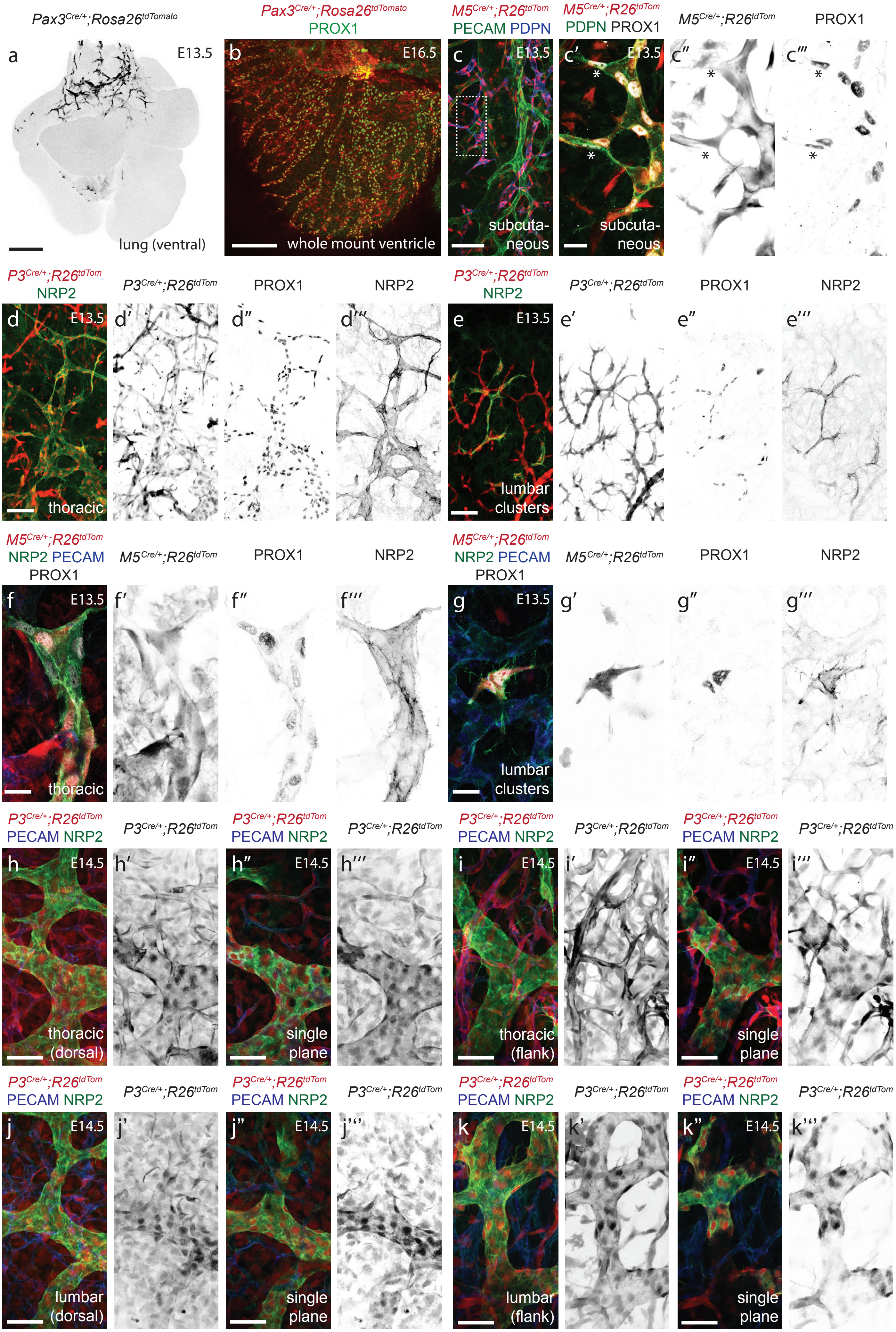

Pax3Cre/+;Rosa26tdTomato
a E13.5
lung (ventral)
Pax3Cre/+;Rosa26tdTomato
PROX1
b E16.5
whole mount ventricle
M5Cre/+;R26tdTom
PECAM PDPN
c E13.5
subcuta-
neous
M5Cre/+;R26tdTom
PDPN PROX1
c′ E13.5
subcuta-
neous
M5Cre/+;R26tdTom
c″
PROX1
c‴
P3Cre/+;R26tdTom
NRP2
d E13.5
thoracic
P3Cre/+;R26tdTom
d′
PROX1
d″
NRP2
d‴
P3Cre/+;R26tdTom
NRP2
e E13.5
lumbar
clusters
P3Cre/+;R26tdTom
e′
PROX1
e″
NRP2
e‴
M5Cre/+;R26tdTom
NRP2 PECAM
PROX1
f E13.5
thoracic
M5Cre/+;R26tdTom
f′
PROX1
f″
NRP2
f‴
M5Cre/+;R26tdTom
NRP2 PECAM
PROX1
g E13.5
lumbar
clusters
M5Cre/+;R26tdTom
g′
PROX1
g″
NRP2
g‴
P3Cre/+;R26tdTom
PECAM NRP2
h E14.5
thoracic
(dorsal)
P3Cre/+;R26tdTom
h′
P3Cre/+;R26tdTom
PECAM NRP2
h″ E14.5
single
plane
P3Cre/+;R26tdTom
h‴
P3Cre/+;R26tdTom
PECAM NRP2
i E14.5
thoracic
(flank)
P3Cre/+;R26tdTom
i′
P3Cre/+;R26tdTom
PECAM NRP2
i″ E14.5
single
plane
P3Cre/+;R26tdTom
i‴
P3Cre/+;R26tdTom
PECAM NRP2
j E14.5
lumbar
(dorsal)
P3Cre/+;R26tdTom
j'
P3Cre/+;R26tdTom
PECAM NRP2
j" E14.5
single
plane
P3Cre/+;R26tdTom
j'''
P3Cre/+;R26tdTom
PECAM NRP2
k E14.5
lumbar
(flank)
P3Cre/+;R26tdTom
k'
P3Cre/+;R26tdTom
PECAM NRP2
k" E14.5
single
plane
P3Cre/+;R26tdTom
k'''

Figure S2: Related to Figure 2. Paraxial mesodermal cells give rise to the subcutaneous and dermal lymphatics.

(**a**) Representative confocal projection of $Pax3^{Cre/+};Rosa26^{tdtomato}$ whole mount embryonic lungs. (**b**) Representative confocal projection of a $Pax3^{Cre/+};Rosa26^{tdtomato}$ whole mount embryonic ventricle co-immunostained for PROX1. (**c**-**c**''') Immunofluorescence staining for tdTomato, PECAM, PDPN and PROX1 on a sagtital vibratome section at the level of the subcutaneous lymphatic network from a $Myf5^{Cre/+};Rosa26^{tdtomato}$ embryo. (**d**-**e**''') Immunofluorescence for tdTomato, NRP2 and PROX1 on whole mount embryonic skin from $Pax3^{Cre/+};Rosa26^{tdtomato}$ embryos at the indicated stage and locations. (**f**-**g**''') Immunofluorescence for tdTomato, NRP2 and PROX1 on whole mount embryonic skin from $Myf5^{Cre/+};Rosa26^{tdtomato}$ embryos at the indicated stage and locations. High resolution and single confocal plane images of immunofluorescence for tdTomato, PECAM and NRP2 on whole mount embryonic skin from $Pax3^{Cre/+};Rosa26^{tdtomato}$ embryos at the indicated stage and locations (**h-k'''**). Scale bars: a, b – 200 µm; c, d, e – 100 µm; c'-c''', f, g – 20 µm; h-k – 50 µm.

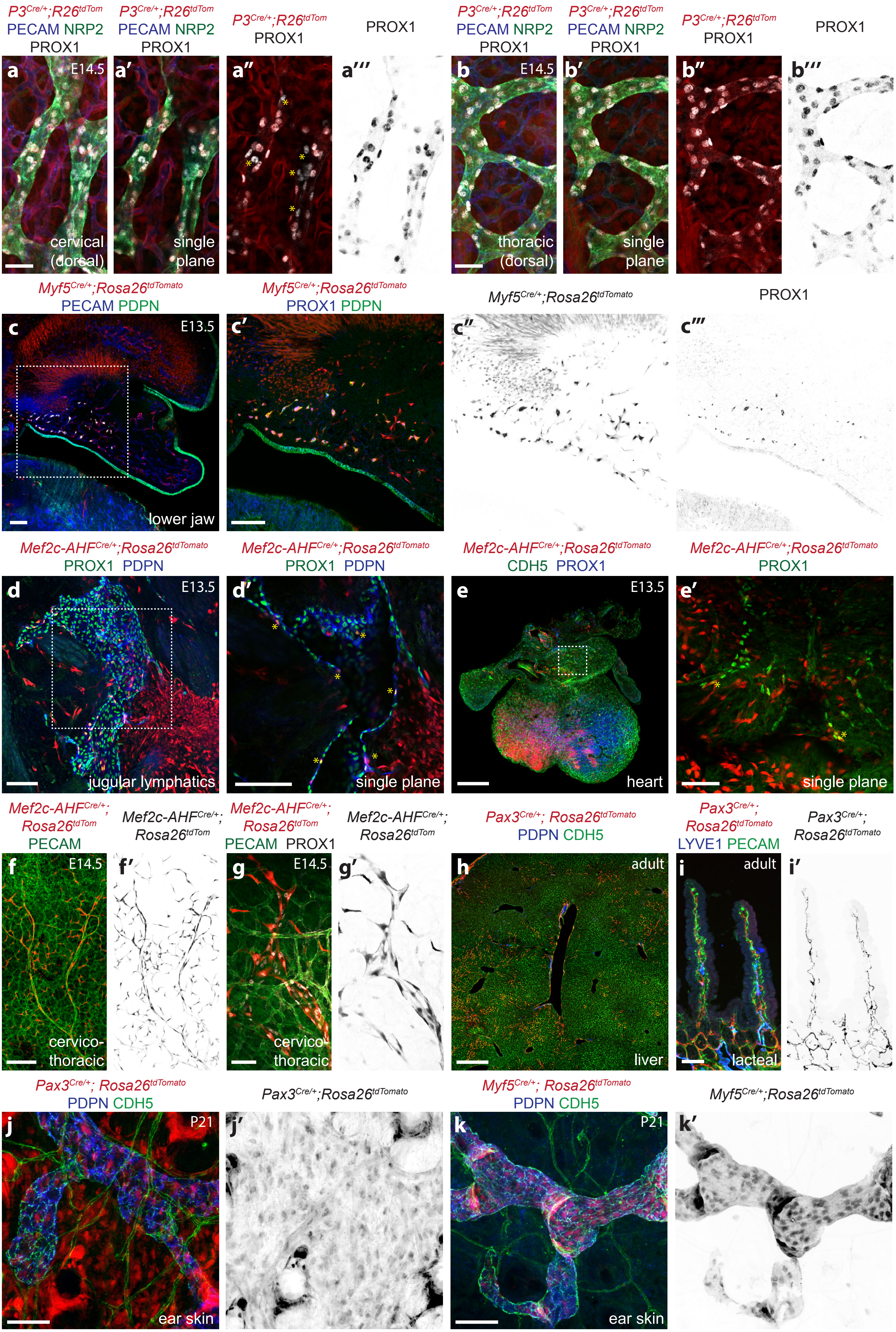

P3Cre/+;R26tdTom PECAM NRP2 PROX1
P3Cre/+;R26tdTom PROX1
PROX1
a
E14.5
cervical (dorsal)
a'
single plane
a''
a'''
b
E14.5
thoracic (dorsal)
b'
single plane
b''
b'''
Myf5Cre/+;Rosa26tdTomato PECAM PDPN
Myf5Cre/+;Rosa26tdTomato PROX1 PDPN
Myf5Cre/+;Rosa26tdTomato
PROX1
c
E13.5
lower jaw
c'
c''
c'''
Mef2c-AHFCre/+;Rosa26tdTomato PROX1 PDPN
Mef2c-AHFCre/+;Rosa26tdTomato CDH5 PROX1
Mef2c-AHFCre/+;Rosa26tdTomato PROX1
d
E13.5
jugular lymphatics
d'
single plane
e
E13.5
heart
e'
single plane
Mef2c-AHFCre/+; Rosa26tdTom PECAM
Mef2c-AHFCre/+; Rosa26tdTom
Mef2c-AHFCre/+; Rosa26tdTom PECAM PROX1
Pax3Cre/+; Rosa26tdTomato PDPN CDH5
Pax3Cre/+; Rosa26tdTomato LYVE1 PECAM
Pax3Cre/+; Rosa26tdTomato
f
E14.5
cervico-thoracic
f'
g
E14.5
cervico-thoracic
g'
h
adult
liver
i
adult
lacteal
i'
Pax3Cre/+; Rosa26tdTomato PDPN CDH5
Pax3Cre/+;Rosa26tdTomato
Myf5Cre/+; Rosa26tdTomato PDPN CDH5
Myf5Cre/+;Rosa26tdTomato
j
P21
ear skin
j'
k
P21
ear skin
k'

Figure S3: Related to Figures 2 and 3. Contribution of multiple muscle progenitor populations to organ and tissue specific lymphatic vascular beds.

(**a**-**b'''**) Immunofluorescence for tdTomato, PECAM, NRP2 and PROX1 on whole mount embryonic skin from *Pax3*$^{Cre/+}$*;Rosa26*^{tdtomato} embryos at the indicated stage and locations . Yellow asterisks highlight PROX1^{+} cells that are not labeled by *Pax3*$^{Cre/+}$*;Rosa26*^{tdtomato} in the cervical skin. (**c-c'''**) Immunofluorescence for tdTomato, PECAM, PDPN and PROX1 on sagittal vibratome sections at the level of the lower jaw from *Myf5*$^{Cre/+}$*;Rosa26*^{tdTomato} embryos. (**d-d'**) Immunofluorescence for tdTomato, PROX1 and PDPN on sagittal vibratome sections at the level of the jugular lymph sac from *Mef2c-AHF*$^{Cre/+}$*;Rosa26*^{tdTomato} embryos - ellow asterisks indicate LECs derived from the *Mef2c-AHF* lineage. (**e-e'**) Representative confocal images of a *Mef2c-AHF*$^{Cre/+}$*;Rosa26*^{tdtomato} whole mount embryonic heart, co-immunostained for CDH5 and PROX1 - yellow asterisks indicate LECs derived from the *Mef2c-AHF* lineage. Immunofluorescence for tdTomato and PECAM (**f-f'**), and tdTomato, PECAM and PROX1 (**g-g'**) on whole mount embryonic cervico-thoracic skin from *Mef2c-AHF*$^{Cre/+}$*;Rosa26*^{tdtomato} embryos. (**h**) Immunofluorescence for tdTomato, PDPN and CDH5 on cryosectioned liver tissue from an adult *Pax3*$^{Cre/+}$*;Rosa26*^{tdtomato} mouse. (**i-i'**) Immunofluorescence for tdTomato, LYVE1 and PECAM on cryosectioned jejunum from an adult *Pax3*$^{Cre/+}$*;Rosa26*^{tdtomato} mouse. Immunofluorescence for tdTomato, PDPN and CDH5 on whole mount ear skin from *Pax3*$^{Cre/+}$*;Rosa26*^{tdtomato} (**j**, **j'**) or *Myf5*$^{Cre/+}$*;Rosa26*^{tdtomato} (**k**, **k'**) mice at post-natal day 21. Scale bars: a-c''', e', g-g' – 50 µm; d-d', g-g', i-i', j-k' – 100 µm; e – 1 mm; f-f' – 200 µm; h – 500 µm.

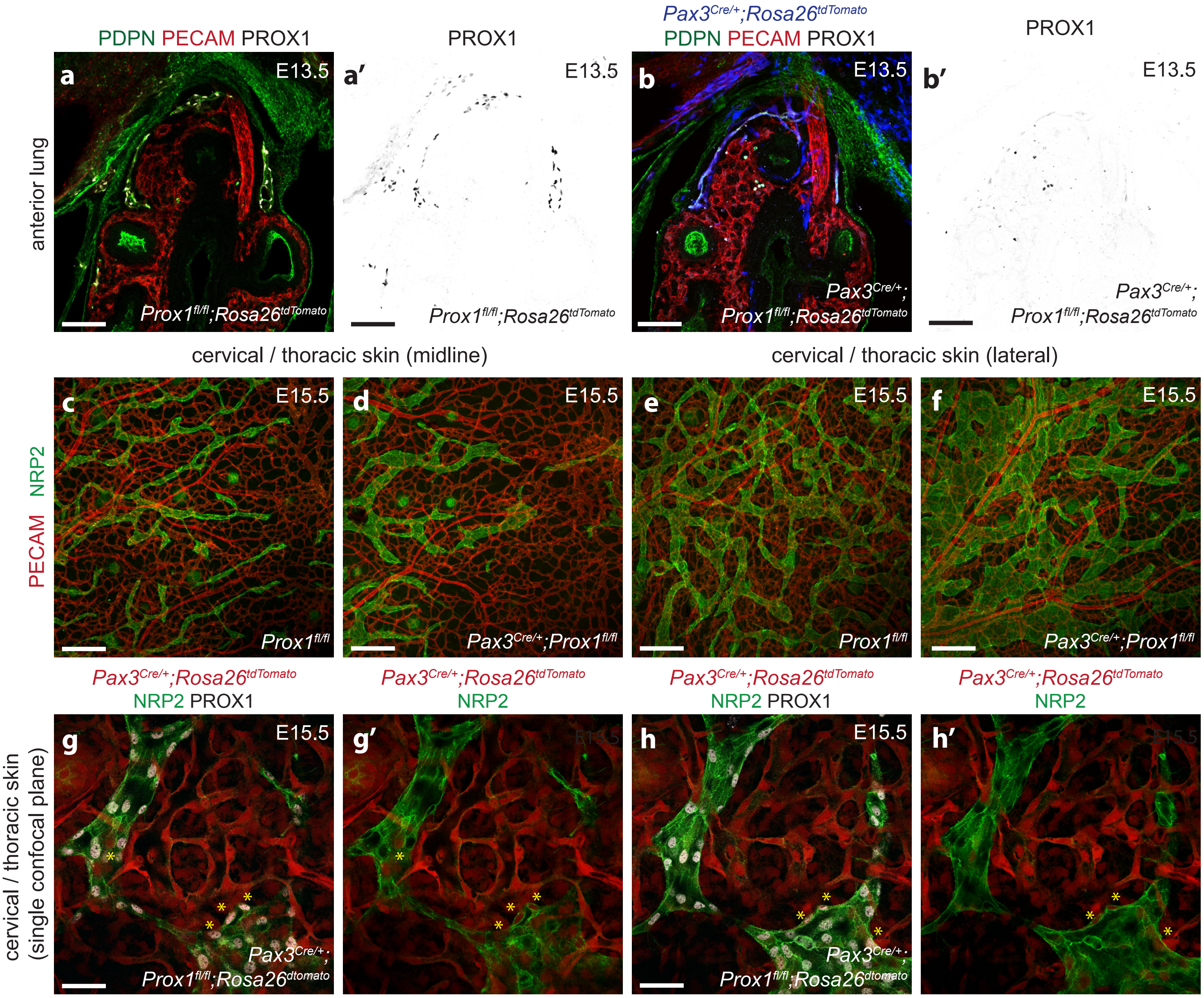

PDPN PECAM PROX1
PROX1
Pax3Cre/+;Rosa26tdTomato
PDPN PECAM PROX1
PROX1
anterior lung
a
E13.5
Prox1fl/fl;Rosa26tdTomato
a′
E13.5
Prox1fl/fl;Rosa26tdTomato
b
E13.5
Pax3Cre/+;
Prox1fl/fl;Rosa26tdTomato
b′
E13.5
Pax3Cre/+;
Prox1fl/fl;Rosa26tdTomato
cervical / thoracic skin (midline)
cervical / thoracic skin (lateral)
PECAM NRP2
c
E15.5
Prox1fl/fl
d
E15.5
Pax3Cre/+;Prox1fl/fl
e
E15.5
Prox1fl/fl
f
E15.5
Pax3Cre/+;Prox1fl/fl
Pax3Cre/+;Rosa26tdTomato
NRP2 PROX1
Pax3Cre/+;Rosa26tdTomato
NRP2
Pax3Cre/+;Rosa26tdTomato
NRP2 PROX1
Pax3Cre/+;Rosa26tdTomato
NRP2
cervical / thoracic skin
(single confocal plane)
g
E15.5
Pax3Cre/+;
Prox1fl/fl;Rosa26dtomato
g′
h
E15.5
Pax3Cre/+;
Prox1fl/fl;Rosa26dtomato
h′

Figure S4: Related to Figure 4. Analysis of *Prox1* deletion in the paraxial mesoderm derived cells.

Immunofluorescence for tdTomato, PDPN, PECAM and PROX1 on sagittal vibratome sections at the level of the cardiac outflow tract from $Prox1^{fl/fl};Rosa26^{tdTomato}$ (**a**-**a'**) and $Pax3^{Cre/+};Prox1^{fl/fl};Rosa26^{tdTomato}$ embryos (**b**-**b'**). Immunofluorescence for PECAM and NRP2 on whole mount cervical/thoracic skin from $Prox1^{fl/fl}$ and $Pax3^{Cre/+};Prox1^{fl/fl}$ embryos, at midline (**c**-**d**) and lateral positions (**e**-**f**). Immunofluorescence for tdTomato, NRP2 and PROX1 on whole mount cervical/thoracic skin from $Pax3^{Cre/+};Prox1^{fl/fl}$ embryos (**g**-**h'**) - yellow asterisks highlight $PROX1^+$ cells that are labeled by $Pax3^{Cre/+}; Rosa26^{tdtomato}$ in the cervical/thoracic skin. Scale bars: a, b, d, e, l, m– 50 µm; c, l-m – 100 µm; h-k – 200 µm.
