# Supplementary material for: Paraxial Mesoderm Is the Major Source of Lymphatic Endothelium
Source: Dev Cell. 2019 Jul 22;50(2):247–255.e3. doi: 10.1016/j.devcel.2019.04.034 (PMC6658618; doi:10.1016/j.devcel.2019.04.034)
Supplement: Document S2. Article plus Supplemental Information [file mmc2.pdf]

Short Article

Developmental Cell

Paraxial Mesoderm Is the Major Source of Lymphatic Endothelium

Graphical Abstract

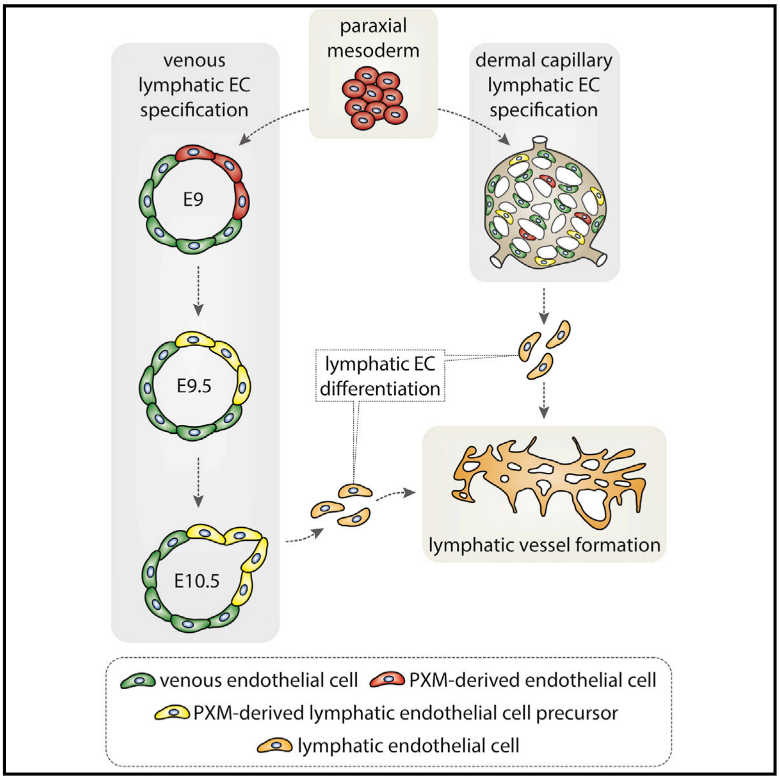

Highlights

- Lineage history is a key determinant of endothelial cell specialization
- Endothelial cells arising from paraxial mesoderm preferentially form lymphatic vessels
- Paraxial-mesoderm-derived endothelial cells are essential for lymphatic development

Authors

Oliver A. Stone, Didier Y.R. Stainier

Correspondence

oliver.stone@dpag.ox.ac.uk (O.A.S.),
didier.stainier@mpi-bn.mpg.de (D.Y.R.S.)

In Brief

Following specification from mesoderm, the prevailing view is that local environmental cues control the specialization of endothelial cells in different vessel beds. Stone and Stainier show that lymphatic endothelial cell fate is imprinted much earlier as cells transition through the paraxial mesoderm.

Stone & Stainier, 2019, Developmental Cell *50*, 247–255
July 22, 2019 © 2019 The Authors. Published by Elsevier Inc.
https://doi.org/10.1016/j.devcel.2019.04.034

Developmental Cell
Short Article

Paraxial Mesoderm Is the Major Source of Lymphatic Endothelium

Oliver A. Stone[1,2,*] and Didier Y.R. Stainier[1,3,*]
[1]Department of Developmental Genetics, Max Planck Institute for Heart and Lung Research, Ludwigstrasse 43, Bad Nauheim 61231, Germany
[2]Department of Physiology, Anatomy and Genetics, BHF Centre of Research Excellence, University of Oxford, Oxford OX1 3PT, UK
[3]Lead Contact
*Correspondence: oliver.stone@dpag.ox.ac.uk (O.A.S.), didier.stainier@mpi-bn.mpg.de (D.Y.R.S.)
https://doi.org/10.1016/j.devcel.2019.04.034

SUMMARY

Endothelial cells (ECs), which line blood and lymphatic vessels, are generally described to come from the lateral plate mesoderm despite experimental evidence for a broader source of origin, including the paraxial mesoderm (PXM). Current dogma suggests that following specification from mesoderm, local environmental cues establish the distinct molecular and functional characteristics of ECs in different vascular beds. Here we present evidence to challenge this view, showing that lymphatic EC fate is imprinted during transition through the PXM lineage. We show that PXM-derived cells form the lymphatic endothelium of multiple organs and tissues, with a more restricted contribution to blood vessel endothelium. By deleting *Prox1* specifically in PXM-derived cells, we show that this lineage is indispensable for lymphatic vessel development. Collectively, our data establish lineage history as a critical determinant of EC specialization, a finding with broad implications for our understanding of vascular development and heterogeneity.

INTRODUCTION

During the iterative process of differentiation, intrinsic and extrinsic cues establish heterogeneity at the cell, organ, and system level. A striking paradigm for this diversification can be observed in the endothelium, which differentiates from mesoderm to form arterial, venous, lymphatic, and organ-specific vessel networks (Herbert and Stainier, 2011). Although multiple cellular origins for endothelial cells (ECs) have been described (Plein et al., 2018; Reischauer et al., 2016; Klotz et al., 2015; Stanczuk et al., 2015; Nguyen et al., 2014; Wilting et al., 1995), the impact of cell lineage on EC diversification is poorly understood. The prevailing view is that tissue-derived signals, coupled to vessel subtype-specific transcriptional networks, establish molecular and functional heterogeneity as ECs invade different organs and tissues (Potente and Mäkinen, 2017).

The initial steps of vascular development take place during gastrulation as mesodermal progenitors commit to an endothelial fate. In zebrafish (Reischauer et al., 2016; Mosimann et al., 2015; Nguyen et al., 2014), chick (Wilting et al., 2000; Pardanaud et al., 1996; Couly et al., 1995; Wilting et al., 1995), and mouse (Mayeuf-Louchart et al., 2014; Hutcheson et al., 2009; Wasteson et al., 2008), ECs have been shown to derive from both lateral plate mesoderm (LPM) and paraxial mesoderm (PXM, also known as presomitic mesoderm) (Figure 1A). Additionally, erythro-myeloid progenitor cells have been reported as a source of both yolk sac and intraembryonic ECs in mouse (Plein et al., 2018). Whether ECs derived from these various lineages are functionally distinct, or preferentially contribute to different vascular beds is poorly understood. Intriguingly, transplantation experiments in avian embryos showed that while PXM-derived ECs contribute to the cardinal vein, wing bud, and perineural vascular plexus, they are unable to invade the visceral organs and are excluded from the ventral wall of the dorsal aorta (Pardanaud et al., 1996), suggesting that ECs from divergent sources may possess unique functional and molecular properties.

The cellular origin of lymphatic vessels has been the subject of debate for over a century (Ulvmar and Mäkinen, 2016; Srinivasan et al., 2007; Huntington and McClure, 1910; Sabin, 1902). Live imaging in zebrafish showed that lymphatic ECs (LECs) arise from venous endothelium (Yaniv et al., 2006), and lineage tracing analyses in mouse have shown that the majority of LECs are derived from transdifferentiation of venous (Srinivasan et al., 2007), intersegmental (Yang et al., 2012), and capillary (Pichol-Thievend et al., 2018) blood ECs. Additionally, contribution of hemogenic endothelium-derived cells to lymphatics in the mesentery (Stanczuk et al., 2015) and heart (Klotz et al., 2015) has been reported in mouse. Furthermore, studies in *Xenopus* (Ny et al., 2005) and avian models (Wilting et al., 2006; Schneider et al., 1999) have suggested alternative nonendothelial sources of LECs, including the PXM.

Following specification from the anterior primitive streak, the PXM extends bilaterally along the anterior-posterior axis adjacent to the neural tube (Hubaud and Pourquié, 2014). Segmentation of the PXM creates the somites, transient structures that contain progenitors for a number of tissues including bone, dorsal dermis, skeletal muscle, and the endothelium of the limb (Buckingham and Rigby, 2014). In chick, clonal analyses of individual somitic cells identified bipotent precursors for the skeletal muscle and endothelium of the limb (Kardon et al., 2002), while cells coexpressing PAX3 (a marker of myogenic precursors) and VEGFR2 (vascular endothelial growth factor receptor 2) have been observed in the somite of the mouse

This is an open access article under the CC BY license (http://creativecommons.org/licenses/by/4.0/).

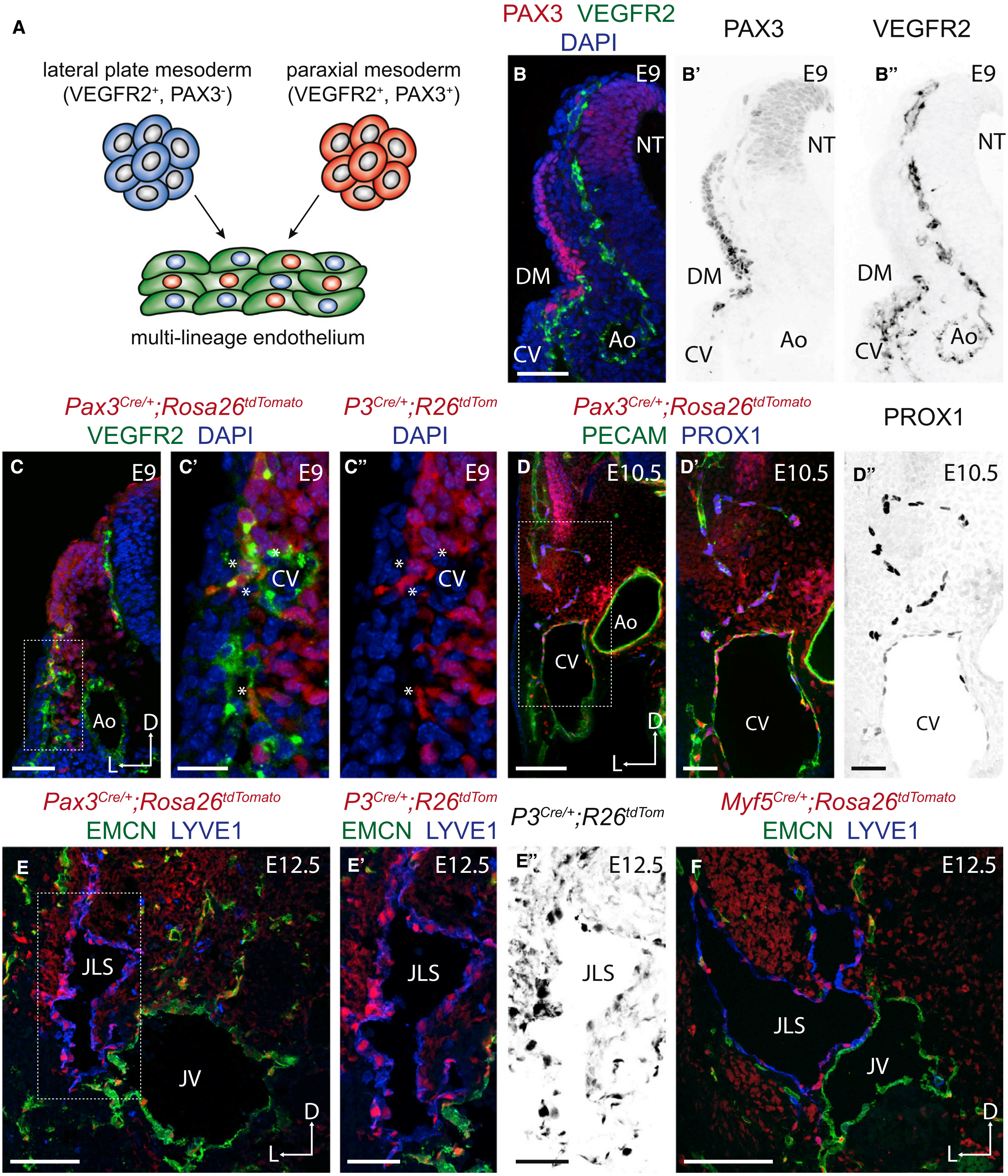

Figure 1. Paraxial Mesodermal Cells Contribute to the Cardinal Vein and Developing Lymphatics

(A) Schematic representation of the contribution of lateral plate and paraxial mesoderm-derived cells to the developing endothelium.

(B–B″) Immunofluorescence for PAX3 and VEGFR2 on transverse cryosections.

(C–C″) Immunofluorescence for tdTomato and VEGFR2 on transverse cryosections from a *Pax3*$^{Cre/+}$;*Rosa26*^{tdtomato} embryo.

(D–D″) Immunofluorescence for tdTomato, PECAM, and PROX1 on transverse vibratome sections from a *Pax3*$^{Cre/+}$;*Rosa26*^{tdtomato} embryo.

(legend continued on next page)

(Mayeuf-Louchart et al., 2014; Kardon et al., 2002). Genetic lineage tracing using Cre recombinase alleles driven from the endogenous transcription start site of the *Pax3* and *Myf5* (myogenic factor 5) genes demonstrated a common origin for skeletal muscle and a subset of the endothelium in the mouse limb (Hutcheson et al., 2009). Furthermore, the descendants of VEGFR2-expressing cells contribute to both endothelium and skeletal muscle in mouse (Motoike et al., 2003). Collectively, these data reveal the existence of a common bipotent progenitor for skeletal muscle and endothelium.

Here, we systematically analyzed the contribution of PXM-derived cells (PXMDCs) to the endothelium of different vascular networks to determine the impact of lineage history on the terminal fate of ECs. Using Cre-loxP-based lineage tracing and high-resolution imaging, these analyses have revealed that PXMDCs are preferentially localized to the dorsal aspect of the cardinal vein before the expression of the earliest known markers of lymphatic differentiation. PXM-derived ECs selectively transdifferentiate from the cardinal vein to form LEC progenitors and subsequently give rise to the lymphatic endothelium of multiple organs and tissues, including the cardiopulmonary system, skin and liver, as well as capillary-derived LEC clusters in the embryonic skin. In addition to *Pax3*-lineage somitic muscle progenitors, we also identified a contribution of *Myf5*-lineage cells to lymphatics in the meninges and postnatal skin of the ear, and revealed the anterior second heart field (the cellular origin of a subset of head and neck muscles) as the source of a limited number of anterior jugular lymph sac, cardiac, and dermal LECs. Furthermore, by deleting *Prox1* specifically in the PXM, we find this to be the only lineage competent for LEC differentiation. Collectively, our analyses identify the earliest step in the formation of the lymphatic vasculature and reveal a common progenitor for skeletal muscle and lymphatic endothelium.

RESULTS

Paraxial Mesodermal Cells Contribute to the Cardinal Vein and Developing Lymphatics

Genetic lineage tracing has demonstrated the presence of a multipotent progenitor population in the mouse dermomyotome (a subset of the somitic PXM) that is marked by the expression of PAX3 and MYF5 (Figure S1A), and gives rise to skeletal muscle and endothelium (Buckingham and Rigby, 2014). To examine their contribution to different vascular beds, we lineage traced PXMDCs using established Cre driver lines ($Pax3^{Cre}$ [Engleka et al., 2005], $Pax7^{Cre}$ [Keller et al., 2004], $Myf5^{Cre}$ [Tallquist et al., 2000]) and a ubiquitously expressed lineage reporter ($Rosa26^{tdTomato}$ [Madisen et al., 2010]) (Figures S1B and S1C). We found that PXMDCs contribute to the endothelium of the embryonic forelimb (Figures S1D and S1D′) and gastrocnemius muscle of the adult hindlimb (Figures S1E and S1E′) confirming previous observations (Mayeuf-Louchart et al., 2014; Hutcheson et al., 2009). Analysis of transverse sections from $Pax3^{Cre}$;$Rosa26^{tdTomato}$ embryos at E9 revealed contribution of PXMDCs to the endothelium of the perineural vascular plexus, intersegmental vessels, and the dorsolateral wall of the developing cardinal vein (Figures 1C–1C″, S1F, and S1F′). LEC progenitors are known to differentiate from the dorsolateral wall of the cardinal vein under the transcriptional control of SOX18, COUPTFII, and PROX1 (François et al., 2008; Srinivasan et al., 2007; You et al., 2005; Wigle and Oliver, 1999), and previous studies have reported a contribution of somitic cells to the lymphatic endothelium in avian embryos (Wilting et al., 2006; He et al., 2003; Schneider et al., 1999). Therefore, we investigated whether PXMDCs contribute to the lymphatic endothelium using immunofluorescence for established LEC markers. Analysis of transverse sections from $Pax3^{Cre}$;$Rosa26^{tdTomato}$ embryos at E10.5 demonstrated that PROX1-positive LEC progenitors located within and migrating from the cardinal vein were derived from the PXM (Figures 1D–1D″). Subsequently, we found these cells migrating from the cardinal vein to form the jugular lymph sacs (JLS) (Figures 1E–1E″). To determine the spatiotemporal pattern of differentiation from the dermomyotome, we lineage traced PXMDCs using $Pax7^{Cre}$ (restricted to the central dermomyotome; Figure S1A) and $Myf5^{Cre}$ (later onset of expression during myogenic lineage commitment; Figure S1B) lines. Consistent with a previous report (Hutcheson et al., 2009), we observed contribution of the *Myf5* (Figure 1F), but not the *Pax7* lineage (Figures S1G and S1H), to the endothelium. *Myf5* lineage cells also contributed to the embryonic lymph sacs, albeit to a lesser extent than *Pax3* lineage cells (Figure 1F), indicating that commitment of PXMDC to the lymphatic endothelium is initiated before the onset of *Myf5* expression in the dermomyotome. Collectively, these data indicate that LEC fate may become hard wired as cells transition through the PXM lineage.

Cardiopulmonary, Subcutaneous, and Dermal Lymphatics Are Derived from the Paraxial Mesoderm

To determine their contribution to the endothelium of different lymphatic beds, we lineage traced PXMDCs and analyzed whole-mount tissues and sections at various embryonic stages. Sagittal vibratome sections of E13.5 $Pax3^{Cre}$;$Rosa26^{tdTomato}$ embryos immunostained for PROX1 and PECAM revealed labeling of LECs migrating into the cardiopulmonary system with limited or no contribution to blood ECs (BECs) (Figures 2A–2A″). PXMDCs initially migrate onto the ventral side of the embryonic lung (Figure S2A). Imaging of fetal and adult hearts revealed that most cardiac LECs were labeled in $Pax3^{Cre}$;$Rosa26^{tdTomato}$ animals (Figure S2B), reflecting the migration of PXMDCs from the venous endothelium, which is the source of most cardiac LECs (Klotz et al., 2015). The lymphatic vasculature of the embryonic skin has been shown to form through lymphangiogenic sprouting from the JLS in the cervicothoracic region and differentiation from the dermal capillary plexus in the lumbar dermis and the cervicothoracic midline (Pichol-Thievend

(E–E″) Immunofluorescence for tdTomato, EMCN, and LYVE1 on transverse cryosections from a $Pax3^{Cre/+}$;$Rosa26^{tdtomato}$ embryo.
(F) Immunofluorescence for tdTomato, EMCN, and LYVE1 on transverse cryosections from a $Myf5^{Cre/+}$;$Rosa26^{tdtomato}$ embryo.
(CV, cardinal vein; Ao, aorta; DM, dermomyotome; NT, neural tube; JLS, jugular lymph sac; JV, jugular vein.)
Scale bars: 50 μm (B, C, E′, and E″); 20 μm (C′ and C″); 100 μm (D, E, and F).

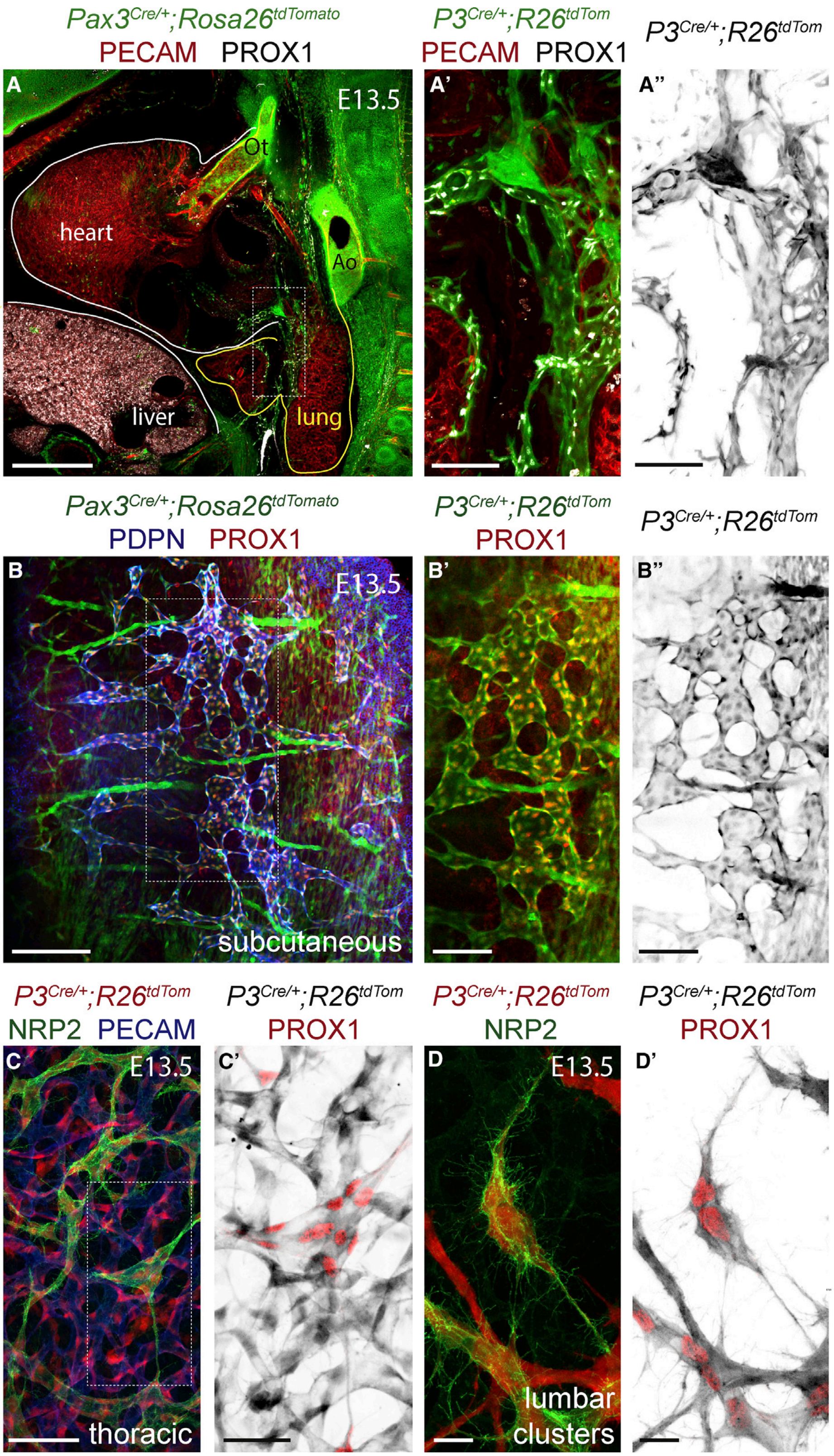

Figure 2. Paraxial Mesodermal Cells Give Rise to the Cardiopulmonary, Subcutaneous, and Dermal Lymphatics

(A–A″) Immunofluorescence for tdTomato, PECAM, and PROX1 on a sagittal vibratome section at the level of the cardiac outflow tract from a *Pax3*$^{Cre/+}$;*Rosa26*^{tdtomato} embryo.

(legend continued on next page)

et al., 2018). Sagittal vibratome sections of E13.5 *Pax3^{Cre};Rosa26^{tdTomato}* (Figures 2B–2B″) and *Myf5^{Cre};Rosa26^{tdTomato}* (Figures S2C–S2C‴) embryos immunostained for podoplanin (PDPN) and PROX1 revealed that subcutaneous LECs spanning the lateral lumbar region are PXM-derived. Analysis of E13.5 whole-mount skin immunostained for PECAM and NRP2 showed that the superficial thoracic dermal LECs (Figures 2C, 2C′, S3D, and S3F), which migrate from the JLS, and the clustered lumbar dermal LECs (Figures 2D, 2D′, S3E, and S3G), which arise from the dermal capillary plexus, are initially derived from the PXM. These data indicate that dermal LECs differentiate from venous and dermal capillary progenitor populations that share a common PXM origin. At later embryonic stages, PXMDCs comprise the lymphatic endothelium of the thoracic, lumbar, and sacral dermis (Figures S3H–S3K‴). However, analysis of whole-mount skin immunostained for PECAM, NRP2, and PROX1 revealed that the lymphatic endothelium of the cervical skin is a mixture of cells from *Pax3^{+ve}* and *Pax3^{-ve}* lineages (Figures S3A–S3B‴).

Distinct Muscle Progenitor Populations Give Rise to Lymphatics

PAX3 labels myogenic precursors in the somitic PXM, but independent gene regulatory networks control myogenesis in the head and neck (Braun and Gautel, 2011); the contribution of *Pax3* lineage cells to the lymphatic endothelium may be spatially restricted in a similar manner. While the expression of PAX3 is limited to the somitic PXM, myogenic regulatory factors such as MYF5 determine muscle fate throughout the body (Braun and Gautel, 2011). Analysis of the head lymphatics in E13.5 *Myf5^{Cre};Rosa26^{tdTomato}* embryos revealed a contribution of *Myf5* lineage cells to the lymphatic endothelium of the lower jaw (Figures S3C–S3C‴), a population of LECs that is not labeled in *Pax3^{Cre};Rosa26^{tdTomato}* embryos. To understand whether other populations of muscle progenitors may also serve as LEC progenitors, we next analyzed the *Mef2c-AHF^{Cre}* line, which labels derivatives of the anterior second heart field (Verzi et al., 2005). A subset of the head and neck muscles have been shown to derive from multipotent progenitor cells that also give rise to second heart field-derived regions of the heart (Diogo et al., 2015). These muscles are labeled in *Mef2c-AHF^{Cre}*, but not in *Pax3^{Cre}* mice (Lescroart et al., 2015). Sagittal vibratome sections of E13.5 *Mef2c-AHF^{Cre};Rosa26^{tdTomato}* embryos stained for PROX1 and PDPN revealed labeling of a small number of LECs in the anterior JLS (Figures S3D and S3D′). Furthermore, imaging of embryonic hearts immunostained for CDH5 and PROX1 showed that a minor proportion of ventral cardiac LECs are also labeled in *Mef2c-AHF^{Cre};Rosa26^{tdTomato}* animals (Figures S3E and S3E′). Moreover, analysis of whole-mount skin revealed a contribution of the *Mef2c-AHF* lineage to the cervicothoracic dermal capillary plexus (Figures S3F and S3F′) and lymphatic endothelium (Figures S3G and S3G′). Collectively, these data indicate that LECs may arise from multiple anatomically distinct muscle progenitor populations.

Paraxial Mesoderm-Derived Cells Form Organ-Specific Lymphatic Networks

To determine their contribution to the postnatal and adult lymphatic endothelium, we lineage traced PXMDCs and analyzed whole-mount tissues and sections. Imaging of adult hearts from *Pax3^{Cre};Rosa26^{tdTomato}* animals revealed labeling of a network of vessels at the surface of the heart (Figure 3A). Immunostaining for LYVE1 and PDPN showed that most of cardiac LECs are PXM-derived (Figure 3A′) with no contribution of PXMDCs to the coronary endothelium. Immunostaining of lung sections from *Pax3^{Cre};Rosa26^{tdTomato}* animals showed that PXMDCs give rise to lymphatic vessels that sit proximal to the airways and major vessels (Figures 3B–3B″), with limited contribution to blood vessel endothelium, while analysis of liver tissue from *Pax3^{Cre};Rosa26^{tdTomato}* animals showed that LECs in this tissue are PXM-derived (Figures 3C–3C″), and also revealed a contribution of PXMDCs to the hepatic blood vessel endothelium (Figure S3H). In contrast to all other visceral organs examined, we did not observe a contribution of PXMDCs to the mesenteric or intestinal lymphatics (Figures S3I and S3I′). Immunostaining of whole-mount meninges (Figures 3D–3D″) and ear skin (Figures S3J–S3K′) revealed that the LECs in these lymphatic beds are derived from a *Myf5^{+ve}* muscle progenitor population with no contribution from the *Pax3* lineage.

Transition through the Paraxial Mesodermal Lineage Is Essential for Lymphatic Endothelial Differentiation

To determine the requirement for PXMDCs during the formation of the lymphatic vasculature (i.e., do cells derived from other mesodermal subtypes have the capacity to differentiate into LECs when this process is impaired in PXMDCs), we conditionally deleted the master regulator of LEC fate *Prox1* (Wigle and Oliver, 1999) in the PXM lineage. For these analyses, we crossed a conditional *Prox1^{fl}* allele (Martinez-Corral et al., 2015) with *Pax3^{Cre}* and examined lymphatic development. Gross morphological assessment revealed that homozygous loss of *Prox1* in the PXM lineage leads to subdermal edema and blood-filled lymphatic vessels in the cervical and thoracic skin at E15.5 (Figures 4A and 4B). Detailed morphological analyses of sagittal vibratome sections immunostained for PDPN, PECAM, and PROX1 showed a lack of subcutaneous LECs in the lateral lumbar region of *Pax3^{Cre};Prox1$^{fl/fl}$* embryos at E13.5 (Figures 4C–4D′). Furthermore, analysis of whole-mount skin immunostained for PECAM and NRP2 revealed a complete lack of lumbar dermal lymphatics at E15.5 (Figures 4E–4F′). Conditional knock out of *Prox1* in the *Tie-2* lineage was previously shown to result in incomplete recombination and the presence of a dysmorphic cardiac lymphatic network (Klotz et al., 2015). Analysis of sagittal vibratome sections at the level of the cardiac outflow tract showed the presence of PXM-derived LECs that expressed low levels of PROX1 in the

(B–B″) Immunofluorescence for tdTomato, PECAM, and PROX1 on a sagittal vibratome section at the level of the subcutaneous lymphatic network from a *Pax3$^{Cre/+}$;Rosa26^{tdtomato}* embryo.

(C–D′) Immunofluorescence for tdTomato, NRP2, and PECAM on whole-mount skin from *Pax3$^{Cre/+}$;Rosa26^{tdtomato}* embryos at the indicated positions and stages. (Ao, aorta; Ot, outflow tract.)

Scale bars: 500 μm (A); 200 μm (B); 100 μm (A′ and A″); 50 μm (C); 20 μm (C′–D′).

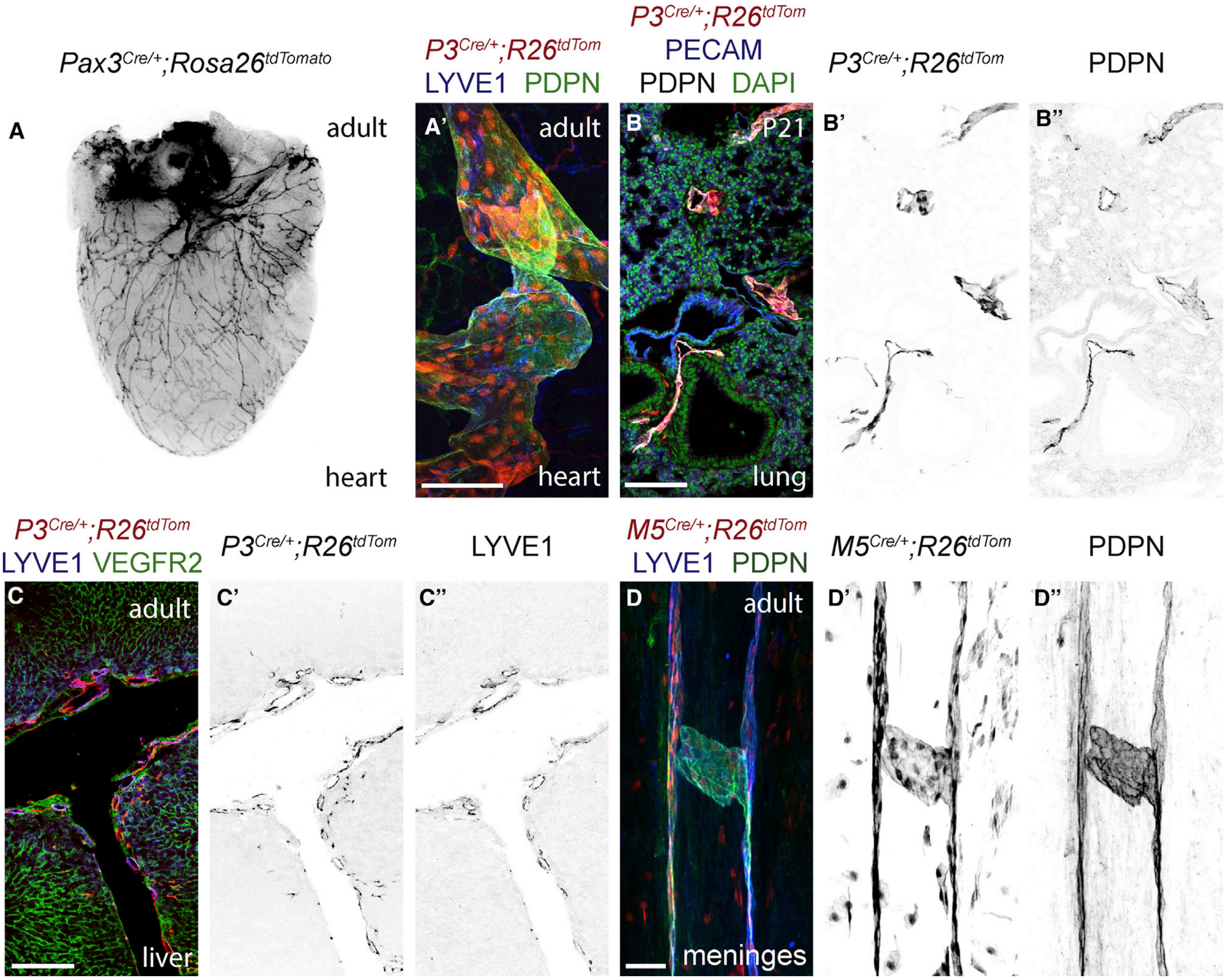

Figure 3. Paraxial Mesoderm-Derived LECs Form the Lymphatic Vasculature of Postnatal and Adult Tissues

(A) Representative whole-mount stereoscopic imaging of the adult heart from a *Pax3$^{Cre/+}$;Rosa26^{tdtomato}* animal.

(A′) Whole-mount immunofluorescence for tdTomato, LYVE1, and PDPN on the adult ventricle from a *Pax3$^{Cre/+}$;Rosa26^{tdtomato}* animal.

(B–B″) Immunofluorescence for tdTomato, PECAM, and PDPN on cryosectioned lung tissue from a postnatal day 21 *Pax3$^{Cre/+}$;Rosa26^{tdtomato}* mouse.

(C–C″) Immunofluorescence for tdTomato, LYVE1, and VEGFR2 on cryosectioned liver tissue from the caudal lobe of a *Pax3$^{Cre/+}$;Rosa26^{tdtomato}* animal.

(D–D″) Whole-mount immunofluorescence for tdTomato, LYVE1, and PDPN on the adult meninges from a *Myf5$^{Cre/+}$;Rosa26^{tdtomato}* animal.

Scale bars: 100 μm (A′ and B–B″); 250 μm (C–C″); 50 μm (D–D″).

anterior region of the lung in *Pax3^{Cre};Prox1$^{fl/fl}$;Rosa26^{tdTomato}* embryos at E13.5 (Figures S4A–S4B′). Furthermore, analysis of whole-mount skin revealed the presence of PROX1-expressing LECs in the thoracic and cervical dermis in *Pax3^{Cre};Prox1$^{fl/fl}$;Rosa26^{tdTomato}* embryos at E15.5 (Figures S4C–S4H′), indicating that recombination of the *Prox1^{fl}* allele is incomplete in certain lymphatic beds. Collectively, these data show an absence of LEC differentiation where complete recombination of *Prox1* is achieved in the PXM lineage, indicating that the PXM may be the only mesodermal subtype competent for LEC differentiation.

DISCUSSION

Our study shows that contrary to current dogma, terminal EC fate is imprinted during mesodermal differentiation and before initiation of the established endothelial genetic program. Akin to the contribution of the first and second heart fields to distinct regions of the heart (Kelly et al., 2014), and the allocation of pre-configured cells to anterior versus posterior fates in the nervous system (Metzis et al., 2018), this work reveals that ECs can be functionally subdivided based on their embryonic origin. In avian embryos, LECs have been shown to derive from both the LPM and PXM (Wilting et al., 2006; Wilting et al., 2000). While accurate lineage tracing of the cranial PXM is currently not possible because of the lack of a distinct morphological and molecular boundary with the cranial LPM (Sambasivan et al., 2011), lineage tracing with *Myf5^{Cre}* indicates that distinct muscle progenitor populations give rise to LECs in the head. Furthermore, lineage tracing with *Mef2c-AHF^{Cre}* identified the second heart field as an additional local progenitor source for LECs in the neck and

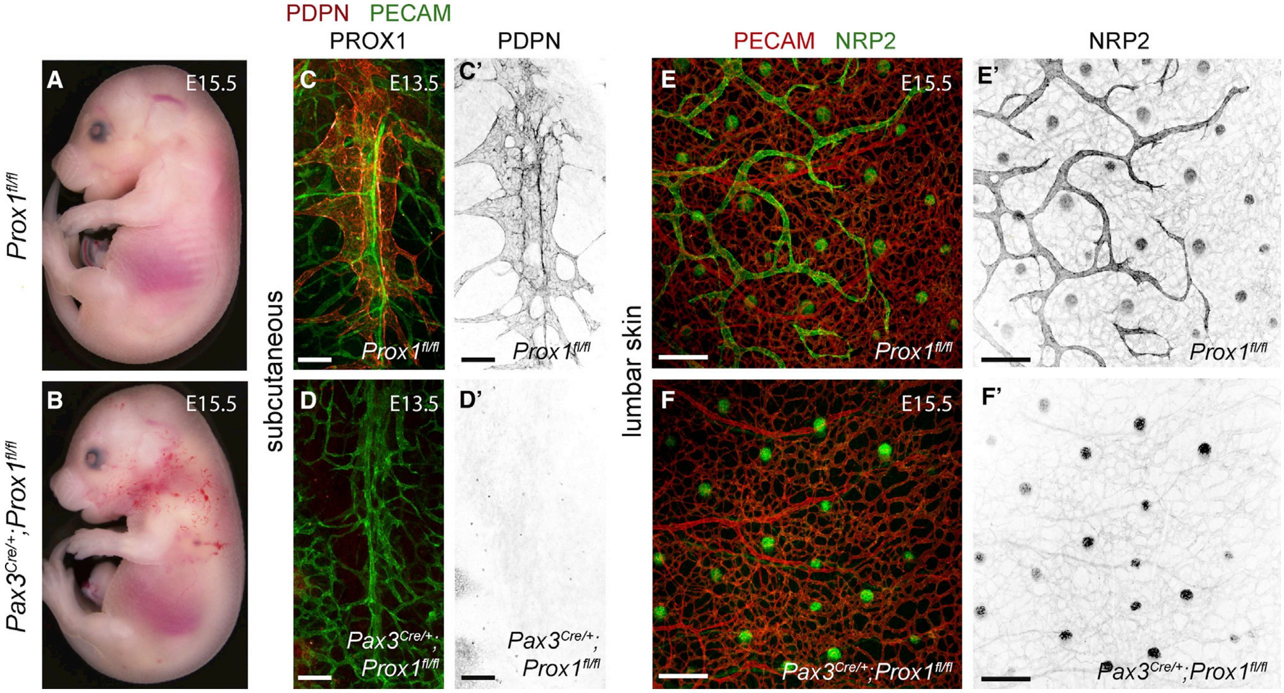

Figure 4. Transition through the Paraxial Mesodermal Lineage Is Essential for LEC Differentiation

Whole-mount brightfield images of *Prox1*$^{fl/fl}$ (A) and *Pax3*$^{Cre/+}$;*Prox1*$^{fl/fl}$ embryos (B) at E15.5. Immunofluorescence for PDPN, PECAM, and PROX1 on sagittal vibratome sections at the level of the subcutaneous lymphatic network from *Prox1*$^{fl/fl}$ (C) and *Pax3*$^{Cre/+}$;*Prox1*$^{fl/fl}$ embryos (D) at E13.5. Immunofluorescence for PECAM and NRP2 on whole-mount lumbar skin from *Prox1*$^{fl/fl}$ (E and E′) and *Pax3*$^{Cre/+}$;*Prox1*$^{fl/fl}$ embryos (F and F′) at E15.5.
Scale bars: 100 μm (C–D′); 200 μm (E–F′).

heart. Collectively, our data indicate that multiple classically defined muscle progenitor populations serve as the cellular source of LECs in distinct anatomical locations.

Our analyses show that LECs in the mouse cardiopulmonary, liver, meningeal, subcutaneous, and dermal lymphatic vessels derive from the PXM lineage, which also makes a more limited and tissue-restricted contribution to the blood vasculature; for example, PXM-derived blood ECs were rarely observed in the heart or lung, but fairly frequently in the liver. A recent study in mouse reported that yolk sac and embryonic erythro-myeloid progenitors are the cellular source of up to 60% of liver ECs (Plein et al., 2018); however, whether erythro-myeloid progenitor-derived ECs are functionally distinct from neighboring ECs was not established. Intriguingly, in an aortic endothelial injury model, a subpopulation of highly proliferative ECs was shown to drive regeneration of the endothelial layer (McDonald et al., 2018), highlighting the functional heterogeneity of neighboring ECs within individual vessels. Future studies comparing PXM-derived ECs with neighboring ECs may reveal important aspects of endothelial heterogeneity that may impact the physiological response of entire vessel networks.

Lymphatic diseases represent a significant healthcare burden with estimates of up to 300 million individuals suffering from primary or secondary lymphedema worldwide (Bellini and Hennekam, 2014). Recent genome-wide analyses have uncovered novel molecular players in the development of primary lymphedema; however, the genetic basis of more than 75% of all cases is currently unknown (Brouillard et al., 2014). Furthermore, current therapies for secondary lymphedema are palliative (Grada and Phillips, 2017). Our findings identify the earliest step in LEC differentiation, which precedes induction of LEC fate by the well-described SOX18-COUPTFII-PROX1 axis (Francois et al., 2011). It is thus likely that investigation of the molecular cues that drive specification of LECs from the PXM will help the discovery of novel causative mutations in primary lymphatic disease in humans. Furthermore, future efforts to define the signals that drive PXM to LEC differentiation *in vivo* may provide a framework to differentiate progenitor cells into LECs, which could be used to treat secondary lymphedema. Notably, as the roles of lymphatics in cancer, cardiovascular disease, immunity, and infection are being described in increasing depth (Mortimer and Rockson, 2014), our findings may also have broad implications for human pathophysiology.

STAR★METHODS

Detailed methods are provided in the online version of this paper and include the following:

- KEY RESOURCES TABLE
- CONTACT FOR REAGENT AND RESOURCE SHARING
- EXPERIMENTAL MODEL AND SUBJECT DETAILS
- METHOD DETAILS
  - Immunostaining and Imaging of Mouse Tissues

SUPPLEMENTAL INFORMATION

Supplemental Information can be found online at https://doi.org/10.1016/j.devcel.2019.04.034.

ACKNOWLEDGMENTS

We thank Taija Mäkinen for *Prox1*^{fl} mice (EUCOMM Consortium), Thomas Braun and Birgit Spitznagel for *Pax3*^{Cre}, *Pax7*^{Cre}, and *Myf5*^{Cre} mice, Sigolène Meilhac for *Mef2c-AHF*^{Cre} mice, Ryota Matsuoka for *Rosa26*^{tdtomato} mice, Sébastien Gauvrit for valuable support, and Thomas Braun, Sébastien Gauvrit, Ines Martinez-Corral, Michael Potente, Paul Riley, Sven Reischauer, and Shuichi Watanabe for discussion. This work was supported by an Excellence Cluster Cardiopulmonary System (ECCPS EXC147, DFG) grant (O.A.S.), the BHF Centre of Research Excellence, Oxford (RE/13/1/30181, O.A.S.), the Max Planck Society (D.Y.R.S.), and the European Research Council (ERC) (D.Y.R.S.).

AUTHOR CONTRIBUTIONS

O.A.S. and D.Y.R.S. conceived the study, interpreted data, and wrote the paper. O.A.S. designed and performed experiments.

DECLARATION OF INTERESTS

The authors declare no competing interests.

Received: July 6, 2018
Revised: March 28, 2019
Accepted: April 19, 2019
Published: May 23, 2019

REFERENCES

Bellini, C., and Hennekam, R.C. (2014). Clinical disorders of primary malfunctioning of the lymphatic system. Adv. Anat. Embryol. Cell Biol. *214*, 187–204.

Braun, T., and Gautel, M. (2011). Transcriptional mechanisms regulating skeletal muscle differentiation, growth and homeostasis. Nat. Rev. Mol. Cell Biol. *12*, 349–361.

Brouillard, P., Boon, L., and Vikkula, M. (2014). Genetics of lymphatic anomalies. J. Clin. Invest. *124*, 898–904.

Buckingham, M., and Rigby, P.W. (2014). Gene regulatory networks and transcriptional mechanisms that control myogenesis. Dev. Cell *28*, 225–238.

Couly, G., Coltey, P., Eichmann, A., and Le Douarin, N.M. (1995). The angiogenic potentials of the cephalic mesoderm and the origin of brain and head blood vessels. Mech. Dev. *53*, 97–112.

Diogo, R., Kelly, R.G., Christiaen, L., Levine, M., Ziermann, J.M., Molnar, J.L., Noden, D.M., and Tzahor, E. (2015). A new heart for a new head in vertebrate cardiopharyngeal evolution. Nature *520*, 466–473.

Engleka, K.A., Gitler, A.D., Zhang, M., Zhou, D.D., High, F.A., and Epstein, J.A. (2005). Insertion of Cre into the Pax3 locus creates a new allele of Splotch and identifies unexpected Pax3 derivatives. Dev. Biol. *280*, 396–406.

François, M., Caprini, A., Hosking, B., Orsenigo, F., Wilhelm, D., Browne, C., Paavonen, K., Karnezis, T., Shayan, R., Downes, M., et al. (2008). Sox18 induces development of the lymphatic vasculature in mice. Nature *456*, 643–647.

Francois, M., Harvey, N.L., and Hogan, B.M. (2011). The transcriptional control of lymphatic vascular development. Physiol. *26*, 146–155.

Grada, A.A., and Phillips, T.J. (2017). Lymphedema: pathophysiology and clinical manifestations. J. Am. Acad. Dermatol. *77*, 1009–1020.

He, L., Papoutsi, M., Huang, R., Tomarev, S.I., Christ, B., Kurz, H., and Wilting, J. (2003). Three different fates of cells migrating from somites into the limb bud. Anat. Embryol. *207*, 29–34.

Herbert, S.P., and Stainier, D.Y. (2011). Molecular control of endothelial cell behaviour during blood vessel morphogenesis. Nat. Rev. Mol. Cell Biol. *12*, 551–564.

Hubaud, A., and Pourquié, O. (2014). Signalling dynamics in vertebrate segmentation. Nat. Rev. Mol. Cell Biol. *15*, 709–721.

Huntington, G.S., and McClure, C.F.W. (1910). The anatomy and development of the jugular lymph sacs in the domestic cat (Felis domestica). Am. J. Anat. *10*, 177–312.

Hutcheson, D.A., Zhao, J., Merrell, A., Haldar, M., and Kardon, G. (2009). Embryonic and fetal limb myogenic cells are derived from developmentally distinct progenitors and have different requirements for beta-catenin. Genes Dev. *23*, 997–1013.

Kardon, G., Heanue, T.A., and Tabin, C.J. (2002). Pax3 and Dach2 positive regulation in the developing somite. Dev. Dyn. *224*, 350–355.

Keller, C., Hansen, M.S., Coffin, C.M., and Capecchi, M.R. (2004). Pax3:Fkhr interferes with embryonic Pax3 and Pax7 function: implications for alveolar rhabdomyosarcoma cell of origin. Genes Dev. *18*, 2608–2613.

Kelly, R.G., Buckingham, M.E., and Moorman, A.F. (2014). Heart fields and cardiac morphogenesis. Cold Spring Harb. Perspect. Med. *4*.

Klotz, L., Norman, S., Vieira, J.M., Masters, M., Rohling, M., Dubé, K.N., Bollini, S., Matsuzaki, F., Carr, C.A., and Riley, P.R. (2015). Cardiac lymphatics are heterogeneous in origin and respond to injury. Nature *522*, 62–67.

Lescroart, F., Hamou, W., Francou, A., Théveniau-Ruissy, M., Kelly, R.G., and Buckingham, M. (2015). Clonal analysis reveals a common origin between nonsomite-derived neck muscles and heart myocardium. Proc. Natl. Acad. Sci. USA *112*, 1446–1451.

Madisen, L., Zwingman, T.A., Sunkin, S.M., Oh, S.W., Zariwala, H.A., Gu, H., Ng, L.L., Palmiter, R.D., Hawrylycz, M.J., Jones, A.R., et al. (2010). A robust and high-throughput Cre reporting and characterization system for the whole mouse brain. Nat. Neurosci. *13*, 133–140.

Martinez-Corral, I., Ulvmar, M.H., Stanczuk, L., Tatin, F., Kizhatil, K., John, S.W., Alitalo, K., Ortega, S., and Makinen, T. (2015). Nonvenous origin of dermal lymphatic vasculature. Circ. Res. *116*, 1649–1654.

Mayeuf-Louchart, A., Lagha, M., Danckaert, A., Rocancourt, D., Relaix, F., Vincent, S.D., and Buckingham, M. (2014). Notch regulation of myogenic versus endothelial fates of cells that migrate from the somite to the limb. Proc. Natl. Acad. Sci. USA *111*, 8844–8849.

McDonald, A.I., Shirali, A.S., Aragón, R., Ma, F., Hernandez, G., Vaughn, D.A., Mack, J.J., Lim, T.Y., Sunshine, H., Zhao, P., et al. (2018). Endothelial regeneration of large vessels is a biphasic process driven by local cells with distinct proliferative capacities. Cell Stem Cell *23*, 210–225.e6.

Metzis, V., Steinhauser, S., Pakanavicius, E., Gouti, M., Stamataki, D., Ivanovitch, K., Watson, T., Rayon, T., Mousavy Gharavy, S.N., Lovell-Badge, R., et al. (2018). Nervous system regionalization entails axial allocation before neural differentiation. Cell *175*, 1105–1118.e17.

Mortimer, P.S., and Rockson, S.G. (2014). New developments in clinical aspects of lymphatic disease. J. Clin. Invest. *124*, 915–921.

Mosimann, C., Panáková, D., Werdich, A.A., Musso, G., Burger, A., Lawson, K.L., Carr, L.A., Nevis, K.R., Sabeh, M.K., Zhou, Y., et al. (2015). Chamber identity programs drive early functional partitioning of the heart. Nat. Commun. *6*, 8146.

Motoike, T., Markham, D.W., Rossant, J., and Sato, T.N. (2003). Evidence for novel fate of Flk1+ progenitor: contribution to muscle lineage. Genesis *35*, 153–159.

Nguyen, P.D., Hollway, G.E., Sonntag, C., Miles, L.B., Hall, T.E., Berger, S., Fernandez, K.J., Gurevich, D.B., Cole, N.J., Alaei, S., et al. (2014). Haematopoietic stem cell induction by somite-derived endothelial cells controlled by meox1. Nature *512*, 314–318.

Ny, A., Koch, M., Schneider, M., Neven, E., Tong, R.T., Maity, S., Fischer, C., Plaisance, S., Lambrechts, D., Héligon, C., et al. (2005). A genetic Xenopus laevis tadpole model to study lymphangiogenesis. Nat. Med. *11*, 998–1004.

Pardanaud, L., Luton, D., Prigent, M., Bourcheix, L.M., Catala, M., and Dieterlen-Lievre, F. (1996). Two distinct endothelial lineages in ontogeny, one of them related to hemopoiesis. Development *122*, 1363–1371.

Pichol-Thievend, C., Betterman, K.L., Liu, X., Ma, W., Skoczylas, R., Lesieur, E., Bos, F.L., Schulte, D., Schulte-Merker, S., Hogan, B.M., et al. (2018). A blood capillary plexus-derived population of progenitor cells contributes to

genesis of the dermal lymphatic vasculature during embryonic development. Development *145*.

Plein, A., Fantin, A., Denti, L., Pollard, J.W., and Ruhrberg, C. (2018). Erythro-myeloid progenitors contribute endothelial cells to blood vessels. Nature *562*, 223–228.

Potente, M., and Mäkinen, T. (2017). Vascular heterogeneity and specialization in development and disease. Nat. Rev. Mol. Cell Biol. *18*, 477–494.

Reischauer, S., Stone, O.A., Villasenor, A., Chi, N., Jin, S.W., Martin, M., Lee, M.T., Fukuda, N., Marass, M., Witty, A., et al. (2016). Cloche is a bHLH-PAS transcription factor that drives haemato-vascular specification. Nature *535*, 294–298.

Sabin, F.R. (1902). On the origin of the lymphatic system from the veins and the development of the lymph hearts and thoracic duct in the pig. Am. J. Anat. *1*, 367–389.

Sambasivan, R., Kuratani, S., and Tajbakhsh, S. (2011). An eye on the head: the development and evolution of craniofacial muscles. Development *138*, 2401–2415.

Schneider, M., Othman-Hassan, K., Christ, B., and Wilting, J. (1999). Lymphangioblasts in the avian wing bud. Dev. Dyn. *216*, 311–319.

Srinivasan, R.S., Dillard, M.E., Lagutin, O.V., Lin, F.J., Tsai, S., Tsai, M.J., Samokhvalov, I.M., and Oliver, G. (2007). Lineage tracing demonstrates the venous origin of the mammalian lymphatic vasculature. Genes Dev. *21*, 2422–2432.

Stanczuk, L., Martinez-Corral, I., Ulvmar, M.H., Zhang, Y., Laviña, B., Fruttiger, M., Adams, R.H., Saur, D., Betsholtz, C., Ortega, S., et al. (2015). cKit lineage hemogenic endothelium-derived cells contribute to mesenteric lymphatic vessels. Cell Rep. *10*, 1708–1721.

Tallquist, M.D., Weismann, K.E., Hellström, M., and Soriano, P. (2000). Early myotome specification regulates PDGFA expression and axial skeleton development. Development *127*, 5059–5070.

Ulvmar, M.H., and Mäkinen, T. (2016). Heterogeneity in the lymphatic vascular system and its origin. Cardiovasc. Res. *111*, 310–321.

Verzi, M.P., McCulley, D.J., De Val, S., Dodou, E., and Black, B.L. (2005). The right ventricle, outflow tract, and ventricular septum comprise a restricted expression domain within the secondary/anterior heart field. Dev. Biol. *287*, 134–145.

Wasteson, P., Johansson, B.R., Jukkola, T., Breuer, S., Akyürek, L.M., Partanen, J., and Lindahl, P. (2008). Developmental origin of smooth muscle cells in the descending aorta in mice. Development *135*, 1823–1832.

Wigle, J.T., and Oliver, G. (1999). Prox1 function is required for the development of the murine lymphatic system. Cell *98*, 769–778.

Wilting, J., Aref, Y., Huang, R., Tomarev, S.I., Schweigerer, L., Christ, B., Valasek, P., and Papoutsi, M. (2006). Dual origin of avian lymphatics. Dev. Biol. *292*, 165–173.

Wilting, J., Brand-Saberi, B., Huang, R., Zhi, Q., Köntges, G., Ordahl, C.P., and Christ, B. (1995). Angiogenic potential of the avian somite. Dev. Dyn. *202*, 165–171.

Wilting, J., Papoutsi, M., Schneider, M., and Christ, B. (2000). The lymphatic endothelium of the avian wing is of somitic origin. Dev. Dyn. *217*, 271–278.

Yang, Y., García-Verdugo, J.M., Soriano-Navarro, M., Srinivasan, R.S., Scallan, J.P., Singh, M.K., Epstein, J.A., and Oliver, G. (2012). Lymphatic endothelial progenitors bud from the cardinal vein and intersomitic vessels in mammalian embryos. Blood *120*, 2340–2348.

Yaniv, K., Isogai, S., Castranova, D., Dye, L., Hitomi, J., and Weinstein, B.M. (2006). Live imaging of lymphatic development in the zebrafish. Nat. Med. *12*, 711–716.

You, L.R., Lin, F.J., Lee, C.T., DeMayo, F.J., Tsai, M.J., and Tsai, S.Y. (2005). Suppression of Notch signalling by the COUP-TFII transcription factor regulates vein identity. Nature *435*, 98–104.

STAR★METHODS

KEY RESOURCES TABLE

REAGENT or RESOURCE	SOURCE	IDENTIFIER
Antibodies		
Rat anti-VEGFR2	BD Pharmingen	Cat# 550549; RRID:AB_2132506
Mouse anti-PAX3	R&D Systems	Cat# MAB2457; RRID:AB_2159398
Rat anti-CD31 (PECAM)	BD Pharmingen	Cat# 553370; RRID:AB_394816
Rabbit anti-PROX1	Reliatech GmbH	Cat# 102-PA32AG
Rabbit anti-PROX1	Proteintech	Cat# 11067–2-AP; RRID:AB_2268804
Rat anti-EMCN	Santa Cruz Biotechnology	Cat# SC-65495; RRID:AB_2100037
Rat anti-LYVE1	Reliatech GmbH	Cat# 103-PA50,
Hamster anti-PDPN	Developmental Studies Hybridoma Bank	RRID:AB_531893
Goat anti-NRP2	R&D Systems	Cat# AF567; RRID:AB_2155253
Rabbit anti-ACTA2	GeneTex	Cat# GTX100034; RRID:AB_1240408
CD144 (CDH5)	BD Pharmingen,	Cat# 555289; RRID:AB_395707
Alexa Fluor® 405, Goat anti-Rabbit IgG (H+L)	Thermo Fisher Scientific	Cat# A-31556; RRID:AB_221605
Alexa Fluor® 488, Goat anti-Rat IgG (H+L)	Thermo Fisher Scientific	Cat# A-11006; RRID:AB_2534074
Alexa Fluor® 488, Goat anti-Hamster IgG (H+L)	Thermo Fisher Scientific	Cat# A-21110; RRID:AB_141509
Alexa Fluor® 488, Goat anti-Mouse IgG2a IgG (H+L)	Thermo Fisher Scientific	Cat# A-21131; RRID:AB_141618
Alexa Fluor® 647, Goat anti-Rabbit IgG (H+L)	Thermo Fisher Scientific	Cat# A-21244; RRID:AB_141663
Alexa Fluor® 647, Goat anti-Rat IgG (H+L)	Thermo Fisher Scientific	Cat# A-21247; RRID:AB_141778
Alexa Fluor® 647, Donkey anti-Goat IgG (H+L)	Thermo Fisher Scientific	Cat# A-21447; RRID:AB_141884
Chemicals, Peptides, and Recombinant Proteins		
DAPI	Sigma	Cat# D954
Paraformaldehyde	Sigma	Cat# P6148
Phosphate-buffered saline tablet	Sigma	Cat# P4417
Triton X-100	Sigma	Cat# X-100
Tween 20	Sigma	Cat# P1379
Bovine Serum Albumin	Sigma	Cat# A9418
Normal Goat Serum	Thermo Fisher Scientific	Cat# 16210072
Vectashield®	Vector Laboratories	Cat# H-1000
Tissue-Tek® O.C.T. compound	VWR	Cat# 25608–930
Experimental models: Organisms/Strains		
Pax3 $^{tm1(cre)Joe}$	Engleka et al., 2005	MGI:3804315
Pax7 $^{tm1(cre)Mrc}$	Keller et al., 2004	MGI:3510832
Myf5 $^{tm3(cre)Sor}$	Tallquist et al., 2000	IMSR_JAX007893
Tg(Mef2c-cre)2Blk	Verzi et al., 2005	MGI:3639735
Gt(ROSA)26Sor $^{tm9(CAG-tdTomato)Hze}$	Madisen et al., 2010	IMSR_JAX007909
Prox1 ^{tm1a}*(EUCOMM)* ^{Wtsi}	Martinez-Corral et al., 2015	MGI:5617984
Oligonucleotides		
Pax3 Fw	CTGCACTCGGTGTCACG	*Pax3* ^{Cre} genotyping
Pax3 mut Rev	AGGCAAATTTTGGTGTACGG	
Pax3 wt Rev	AAGCGAGCACAGTGCGGC	
Pax7 wt Fw_1	CTCCTCCACATTCCTTGCTC	*Pax7* ^{Cre} genotyping
Pax7 wt Fw_2	CGGCCTTCTTCTAGGTTCTG	
Pax7 mut Rev_1	GCGGTCTGGCAGTAAAAACTATC	

(*Continued on next page*)

Continued

REAGENT or RESOURCE	SOURCE	IDENTIFIER
Pax7 mut Rev_2	GTGAAACAGCATTGCTGTCACTT	
Myf5 Fw	AACCAGAGACTCCCCAAGGT	*Myf5*^{Cre} genotyping
Myf5 wt Rev	CGGCTCTTAAAGCAATGGTC	
Myf5 mut Rev	ACGAAGTTATTAGGTCCCTCGAC	
Cre Fw	ATTGCTGTCACTTGGTCGTGGC	*Mef2c-AHF*^{Cre} genotyping
Cre Rev	GGAAAATGCTTCTGTCCGTTTGC	*Mef2c-AHF*^{Cre} genotyping
Rosa26tdtomato wt Fw	AAGGGAGCTGCAGTGGAGTA	*Rosa26*^{tdTomato} genotyping
Rosa26tdtomato wt Rev	CCGAAAATCTGTGGGAAGTC	
Rosa26tdtomato mut Fw	GGCATTAAAGCAGCGTATCC	
Rosa26tdtomato mut Rev	CTGTTCCTGTACGGCATGG	
Prox1 flox (a)	TGCTGAAGATGTTGGTTGCT	*Prox1*^{fl} genotyping
Prox1 flox (b)	GGCTTTTCTGTTGCTGAAGG	
Prox1 flox (c)	CTGAACTGATGGCGAGCTCAGAC	

CONTACT FOR REAGENT AND RESOURCE SHARING

Further information and requests for resources and reagents should be directed to and will be fulfilled by the Lead Contact, Didier Y.R. Stainier (didier.stainier@mpi-bn.mpg.de).

EXPERIMENTAL MODEL AND SUBJECT DETAILS

Animals were maintained under standard conditions and all experiments were conducted in accordance with institutional (MPG) and national (Regierungspraesidium, Darmstadt) ethical and animal welfare guidelines. All mouse lines were used as previously described (*Pax3*^{Cre} (Engleka et al., 2005) (RRID:MGI:3804315), *Pax7*^{Cre} (Keller et al., 2004) (RRID:MGI:3510832), *Myf5*^{Cre} (Tallquist et al., 2000) (RRID:IMSR_JAX007893), *Mef2c-AHF*^{Cre} (Verzi et al., 2005) (MGI:3639735), *Rosa26*^{tdTomato} (Madisen et al., 2010) (RRID:IMSR_JAX007909) and *Prox1*^{fl} (Martinez-Corral et al., 2015) (MGI:5617984)) and maintained on a C57BL/6J background. For embryonic staging, the morning of the vaginal plug was considered E0.5. For analyses at E10.5 and earlier, staging was performed by counting somite pairs, and at E12.5 and later by timing of the vaginal plug.

METHOD DETAILS

Immunostaining and Imaging of Mouse Tissues

The following antibodies were used for immunofluorescence staining of cryosections, vibratome sections and whole mount tissues: VEGFR2 (1:200), PAX3 (1:100), CD31 ((PECAM) 1:100), PROX1 (1:200), PROX1 (1:100), EMCN (1:50), LYVE1 (1:250), PDPN (1:20), NRP2 (1:250), ACTA2 (1:400) and CD144 ((CDH5) 1:100). Alexa Fluor conjugated secondary antibodies (Thermo Fisher) were used at 1:300 in all cases.

For immunofluorescence staining of cryosections, samples were fixed in 4% paraformaldehyde (PFA) overnight at 4°C. Samples were washed in 1X PBS, then cryoprotected in sucrose and mounted in Optimal Cutting Temperature (OCT) compound. 8-10 μm cryosections were cut using a Leica CM1950 cryostat. Sections were blocked (1X PBS containing 0.1% Triton X-100 (PBX), 1% Bovine Serum Albumin (BSA) and 2% Normal Goat Serum (NGS)) for 1 hours at RT and then primary antibodies diluted in blocking buffer were incubated overnight. Following three 10-minute washes in PBX, secondary antibodies diluted in blocking buffer were incubated for 1 hours at RT. Slides were then washed three times (10 min) in PBX and where indicated, samples were counterstained with DAPI for visualization of cell nuclei before mounting with Vectashield® Antifade Mounting Medium (CA, USA).

For immunofluorescence staining of vibratome sections, samples were fixed in 4% PFA overnight at 4°C, washed in 1X PBS and then mounted in 5% low melting temperature agarose. 150-200 μm vibratome sections were cut using a Leica VT1000S. Tissue slices were incubated in blocking buffer (1X PBS containing 0.5% Triton X-100, 0.5% Tween 20, 1% BSA and 3% NGS) for 2 hours at RT and then incubated with primary antibodies diluted in incubation buffer (1X PBS containing 0.25% Triton X-100, 0.25% Tween 20, 0.5% BSA and 1.5% NGS) overnight at 4°C. After primary antibody incubation, tissues were washed 5 times in PBX and then incubated with Alexa Fluor conjugated secondary antibodies for 3 hours at room temperature. Tissues were then washed 5 times in PBX and mounted in Vectashield®.

For whole mount staining of the embryonic skin, tissues were fixed in 4% PFA for 2 hours at RT, washed in 1X PBS and then incubated in blocking solution (1X PBS containing 0.3% Triton X-100 and 3% milk) for 2 hours at RT. Tissues were then incubated with primary antibodies diluted in blocking solution overnight at 4°C. After primary antibody incubation, tissues were washed 5 times in

PBX and then incubated with Alexa Fluor conjugated secondary antibodies for 3 hours at room temperature. Tissues were then washed 5 times in PBX and mounted in Vectashield®.

For whole mount imaging of the heart and lungs, samples were fixed in 4% PFA overnight at 4°C, washed in 1X PBS and then blocked (1X PBS containing 0.1% Triton X-100 (PBX), 1% Bovine Serum Albumin (BSA) and 2% Normal Goat Serum (NGS)) for 3 hours at RT. Samples were incubated overnight at 4°C with primary antibodies diluted in incubation buffer (1X PBS containing 0.25% Triton X-100, 0.25% Tween 20, 0.5% BSA and 1.5% NGS). After primary antibody incubation, tissues were washed 5 times in PBX and then incubated with Alexa Fluor conjugated secondary antibodies overnight at 4°C. Tissues were then washed 5 times in PBX and mounted in 2% low melting temperature agarose for imaging.

For whole mount staining of the meninges, tissues were fixed while still attached to the skull cap in 4% PFA for 4 hours at 4°C. Following dissection of the dura mater/arachnoid from the skull cap, tissues were washed in 1X PBS and then incubated in blocking solution (1X PBS containing 0.1% Triton X-100 (PBX), 1% Bovine Serum Albumin (BSA) and 2% Normal Goat Serum (NGS)) for 3 hours at RT. Tissues were then incubated with primary antibodies diluted in blocking solution overnight at 4°C. After primary antibody incubation, tissues were washed 5 times in PBX and then incubated with Alexa Fluor conjugated secondary antibodies for 3 hours at room temperature. Tissues were then washed 5 times in PBX and mounted on glass slides in Vectashield®. Imaging of immunofluorescence stained tissues was performed using a Zeiss LSM700 upright or LSM800 inverted (RRID:SCR_015963) confocal microscope. Whole mount imaging of freshly dissected embryos at E16.5 and adult hearts was performed using a Nikon SMZ25 stereomicroscope.

Developmental Cell, Volume *50*

Supplemental Information

Paraxial Mesoderm Is the Major Source of Lymphatic Endothelium

Oliver A. Stone and Didier Y.R. Stainier

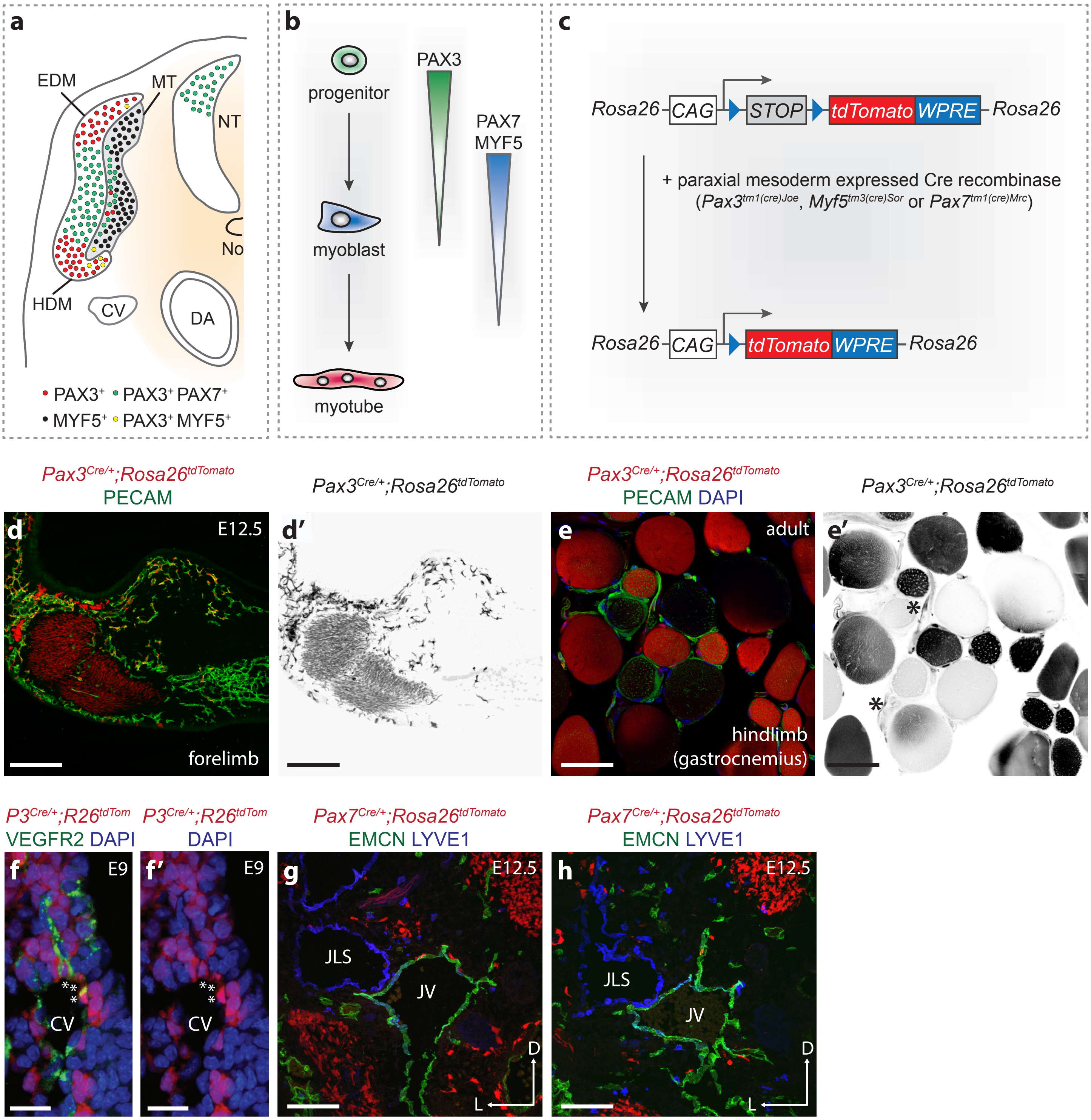

a
EDM
MT
NT
No
HDM
CV
DA
• PAX3+ • PAX3+ PAX7+
• MYF5+ • PAX3+ MYF5+
b
progenitor
PAX3
PAX7
MYF5
myoblast
myotube
c
Rosa26 CAG STOP tdTomato WPRE Rosa26
+ paraxial mesoderm expressed Cre recombinase
(Pax3tm1(cre)Joe, Myf5tm3(cre)Sor or Pax7tm1(cre)Mrc)
Rosa26 CAG tdTomato WPRE Rosa26
Pax3Cre/+;Rosa26tdTomato
PECAM
d
E12.5
forelimb
Pax3Cre/+;Rosa26tdTomato
d'
Pax3Cre/+;Rosa26tdTomato
PECAM DAPI
e
adult
hindlimb
(gastrocnemius)
Pax3Cre/+;Rosa26tdTomato
e'
P3Cre/+;R26tdTom
VEGFR2 DAPI
f
E9
CV
P3Cre/+;R26tdTom
DAPI
f'
E9
CV
Pax7Cre/+;Rosa26tdTomato
EMCN LYVE1
g
E12.5
JLS
JV
D
L
Pax7Cre/+;Rosa26tdTomato
EMCN LYVE1
h
E12.5
JLS
JV
D
L

Figure S1: Related to Figure 1. Analysis of paraxial mesodermal cell contribution to the developing vasculature.

Schematic representations of the embryonic expression of PAX3, PAX7 and MYF5 in the developing dermomyotome, myotome and neural tube (neural crest) (**a**), lineage commitment during embryonic myogenesis (**b**) and the strategy used to lineage trace cells from the paraxial mesoderm (**c**). Immunofluorescence for tdTomato and PECAM on cryosections of embryonic *Pax3*$^{Cre/+}$*;Rosa26*^{tdtomato} forelimb (**d**-**d**') and tdTomato, PECAM and DAPI on cryosections of adult *Pax3*$^{Cre/+}$*;Rosa26*^{tdtomato} gastrocnemius (**e**-**e**'). (**f**-**f**') Immunofluorescence for tdTomato and VEGFR2 on transverse cryosections from a *Pax3*$^{Cre/+}$*;Rosa26*^{tdtomato} embryo. (**g**, **h**) Immunofluorescence for tdTomato, EMCN and LYVE1 on transverse cryosections from *Pax7*$^{Cre/+}$*;Rosa26*^{tdtomato} embryos. (CV - cardinal vein; DA - dorsal aorta; HDM - hypaxial dermomyotome; EDM - epaxial dermomyotome; MT - myotome; No - notochord; NT - neural tube; JLS - jugular lymph sac; JV - jugular vein). Scale bars: **d, d'**, **g**, **h** – 100 µm; **e**-**f**' – 50 µm.

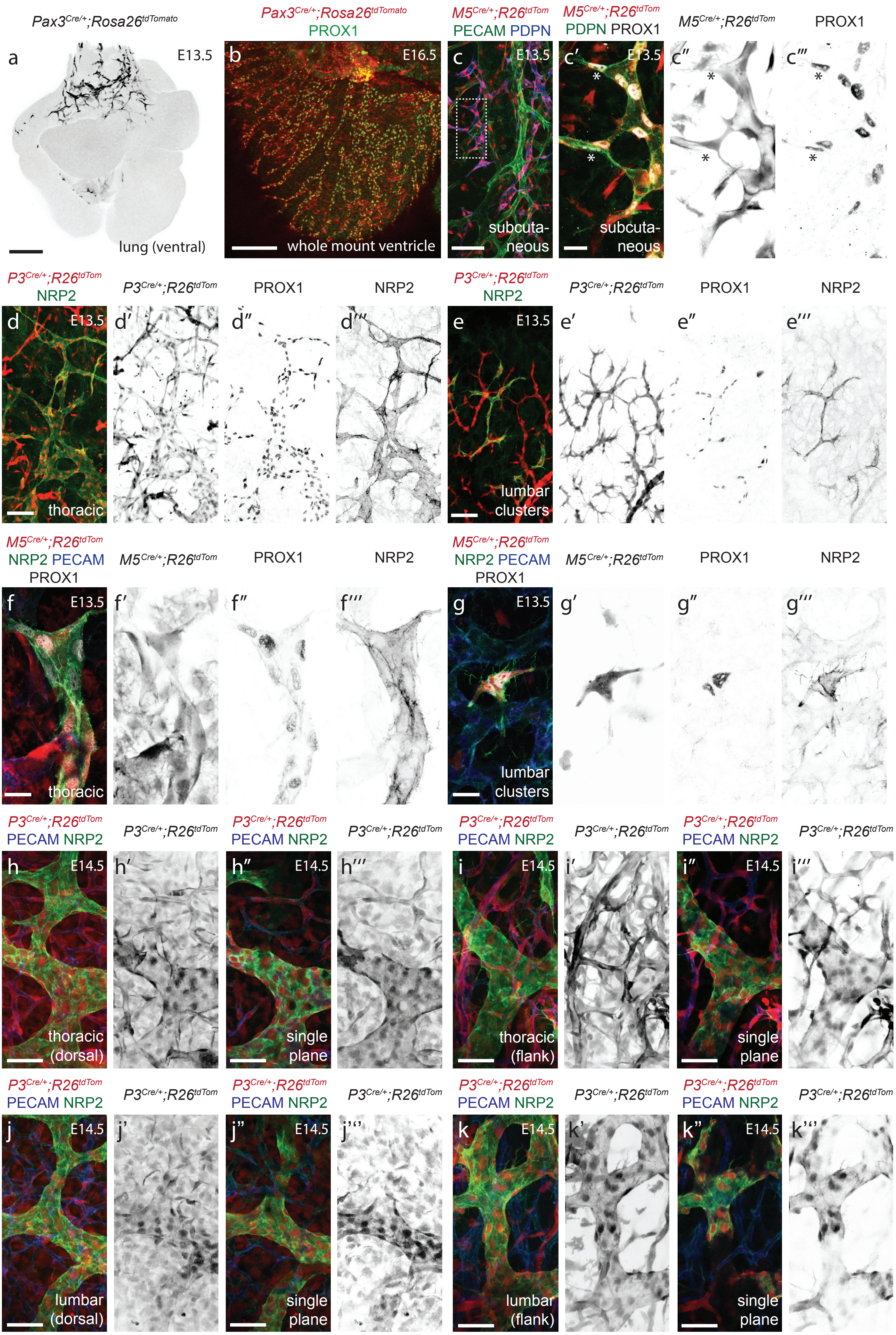

Pax3Cre/+;Rosa26tdTomato
a
E13.5
lung (ventral)
Pax3Cre/+;Rosa26tdTomato
PROX1
b
E16.5
whole mount ventricle
M5Cre/+;R26tdTom
PECAM PDPN
c
E13.5
subcuta-
neous
M5Cre/+;R26tdTom
PDPN PROX1
c′
E13.5
subcuta-
neous
M5Cre/+;R26tdTom
c″
PROX1
c‴
P3Cre/+;R26tdTom
NRP2
d
E13.5
thoracic
P3Cre/+;R26tdTom
d′
PROX1
d″
NRP2
d‴
P3Cre/+;R26tdTom
NRP2
e
E13.5
lumbar
clusters
P3Cre/+;R26tdTom
e′
PROX1
e″
NRP2
e‴
M5Cre/+;R26tdTom
NRP2 PECAM
PROX1
f
E13.5
thoracic
M5Cre/+;R26tdTom
f′
PROX1
f″
NRP2
f‴
M5Cre/+;R26tdTom
NRP2 PECAM
PROX1
g
E13.5
lumbar
clusters
M5Cre/+;R26tdTom
g′
PROX1
g″
NRP2
g‴
P3Cre/+;R26tdTom
PECAM NRP2
h
E14.5
thoracic
(dorsal)
P3Cre/+;R26tdTom
h′
P3Cre/+;R26tdTom
PECAM NRP2
h″
E14.5
single
plane
P3Cre/+;R26tdTom
h‴
P3Cre/+;R26tdTom
PECAM NRP2
i
E14.5
thoracic
(flank)
P3Cre/+;R26tdTom
i′
P3Cre/+;R26tdTom
PECAM NRP2
i″
E14.5
single
plane
P3Cre/+;R26tdTom
i‴
P3Cre/+;R26tdTom
PECAM NRP2
j
E14.5
lumbar
(dorsal)
P3Cre/+;R26tdTom
j’
P3Cre/+;R26tdTom
PECAM NRP2
j”
E14.5
single
plane
P3Cre/+;R26tdTom
j’‘’
P3Cre/+;R26tdTom
PECAM NRP2
k
E14.5
lumbar
(flank)
P3Cre/+;R26tdTom
k’
P3Cre/+;R26tdTom
PECAM NRP2
k”
E14.5
single
plane
P3Cre/+;R26tdTom
k’‘’

Figure S2: Related to Figure 2. Paraxial mesodermal cells give rise to the subcutaneous and dermal lymphatics.

(**a**) Representative confocal projection of *Pax3*$^{Cre/+}$*;Rosa26*^{tdtomato} whole mount embryonic lungs. (**b**) Representative confocal projection of a *Pax3*$^{Cre/+}$*;Rosa26*^{tdtomato} whole mount embryonic ventricle co-immunostained for PROX1. (**c**-**c**''') Immunofluorescence staining for tdTomato, PECAM, PDPN and PROX1 on a sagtital vibratome section at the level of the subcutaneous lymphatic network from a *Myf5*$^{Cre/+}$*;Rosa26*^{tdtomato} embryo. (**d**-**e**''') Immunofluorescence for tdTomato, NRP2 and PROX1 on whole mount embryonic skin from *Pax3*$^{Cre/+}$*;Rosa26*^{tdtomato} embryos at the indicated stage and locations. (**f**-**g**''') Immunofluorescence for tdTomato, NRP2 and PROX1 on whole mount embryonic skin from *Myf5*$^{Cre/+}$*;Rosa26*^{tdtomato} embryos at the indicated stage and locations. High resolution and single confocal plane images of immunofluorescence for tdTomato, PECAM and NRP2 on whole mount embryonic skin from *Pax3*$^{Cre/+}$*;Rosa26*^{tdtomato} embryos at the indicated stage and locations (**h-k'''**). Scale bars: a, b – 200 µm; c, d, e – 100 µm; c'-c''', f, g – 20 µm; h-k – 50 µm.

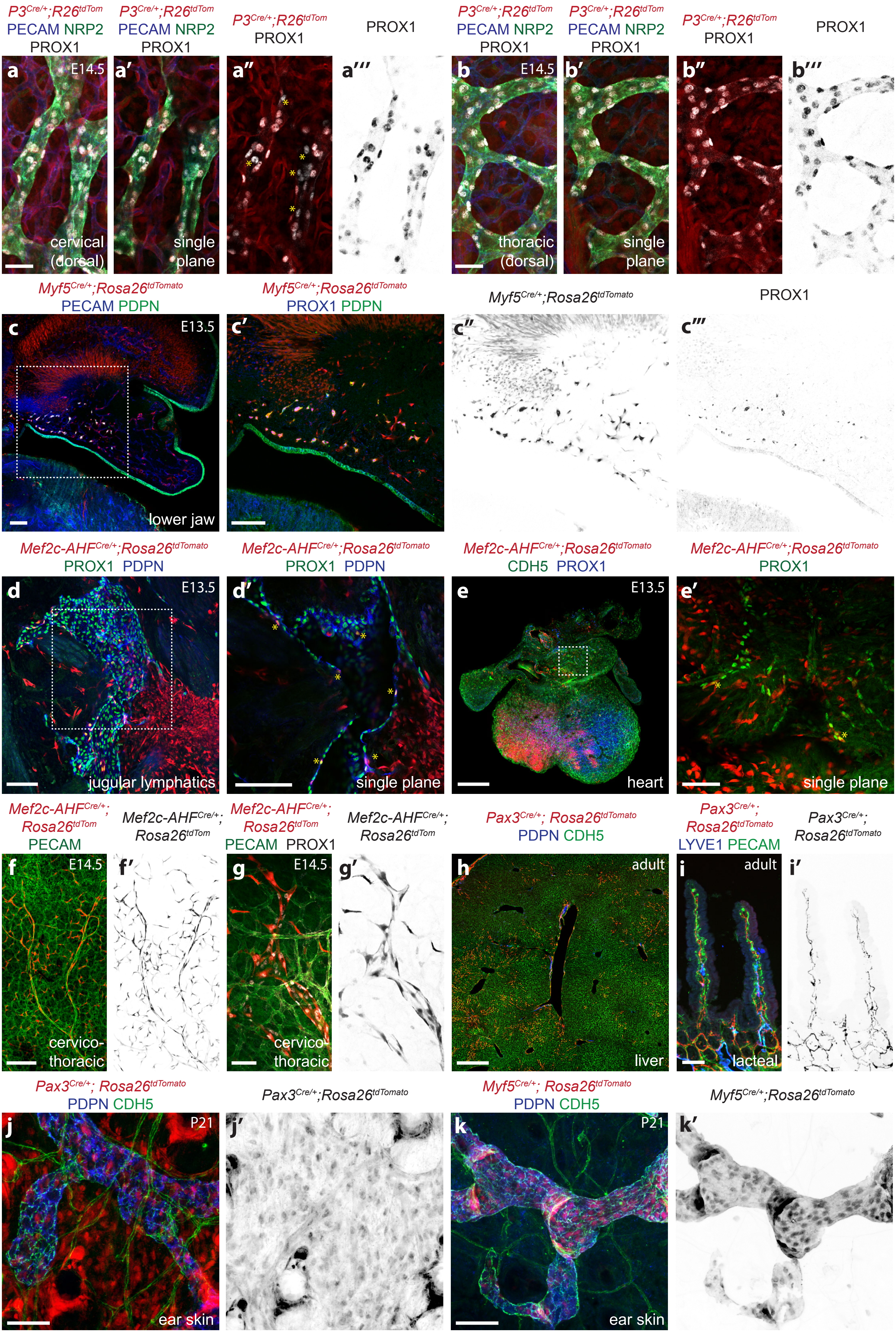

P3Cre/+;R26tdTom PECAM NRP2 PROX1
a E14.5 cervical (dorsal)
a' single plane
P3Cre/+;R26tdTom PROX1
a''
PROX1
a'''
b E14.5 thoracic (dorsal)
b' single plane
b''
b'''
Myf5Cre/+;Rosa26tdTomato PECAM PDPN
c E13.5 lower jaw
Myf5Cre/+;Rosa26tdTomato PROX1 PDPN
c'
Myf5Cre/+;Rosa26tdTomato
c''
PROX1
c'''
Mef2c-AHFCre/+;Rosa26tdTomato PROX1 PDPN
d E13.5 jugular lymphatics
d' single plane
Mef2c-AHFCre/+;Rosa26tdTomato CDH5 PROX1
e E13.5 heart
Mef2c-AHFCre/+;Rosa26tdTomato PROX1
e' single plane
Mef2c-AHFCre/+; Rosa26tdTom PECAM
f E14.5 cervico-thoracic
Mef2c-AHFCre/+; Rosa26tdTom
f'
Mef2c-AHFCre/+; Rosa26tdTom PECAM PROX1
g E14.5 cervico-thoracic
g'
Pax3Cre/+; Rosa26tdTomato PDPN CDH5
h adult liver
Pax3Cre/+; Rosa26tdTomato LYVE1 PECAM
i adult lacteal
Pax3Cre/+; Rosa26tdTomato
i'
Pax3Cre/+; Rosa26tdTomato PDPN CDH5
j P21 ear skin
Pax3Cre/+;Rosa26tdTomato
j'
Myf5Cre/+; Rosa26tdTomato PDPN CDH5
k P21 ear skin
Myf5Cre/+;Rosa26tdTomato
k'

Figure S3: Related to Figures 2 and 3. Contribution of multiple muscle progenitor populations to organ and tissue specific lymphatic vascular beds.

(**a**-**b'''**) Immunofluorescence for tdTomato, PECAM, NRP2 and PROX1 on whole mount embryonic skin from *Pax3*$^{Cre/+}$*;Rosa26*^{tdtomato} embryos at the indicated stage and locations . Yellow asterisks highlight PROX1^{+} cells that are not labeled by *Pax3*$^{Cre/+}$*;Rosa26*^{tdtomato} in the cervical skin. (**c-c'''**) Immunofluorescence for tdTomato, PECAM, PDPN and PROX1 on sagittal vibratome sections at the level of the lower jaw from *Myf5*$^{Cre/+}$*;Rosa26*^{tdTomato} embryos. (**d-d'**) Immunofluorescence for tdTomato, PROX1 and PDPN on sagittal vibratome sections at the level of the jugular lymph sac from *Mef2c-AHF*$^{Cre/+}$*;Rosa26*^{tdTomato} embryos - ellow asterisks indicate LECs derived from the *Mef2c-AHF* lineage. (**e-e'**) Representative confocal images of a *Mef2c-AHF*$^{Cre/+}$*;Rosa26*^{tdtomato} whole mount embryonic heart, co-immunostained for CDH5 and PROX1 - yellow asterisks indicate LECs derived from the *Mef2c-AHF* lineage. Immunofluorescence for tdTomato and PECAM (**f-f'**), and tdTomato, PECAM and PROX1 (**g-g'**) on whole mount embryonic cervico-thoracic skin from *Mef2c-AHF*$^{Cre/+}$*;Rosa26*^{tdtomato} embryos. (**h**) Immunofluorescence for tdTomato, PDPN and CDH5 on cryosectioned liver tissue from an adult *Pax3*$^{Cre/+}$*;Rosa26*^{tdtomato} mouse. (**i-i'**) Immunofluorescence for tdTomato, LYVE1 and PECAM on cryosectioned jejunum from an adult *Pax3*$^{Cre/+}$*;Rosa26*^{tdtomato} mouse. Immunofluorescence for tdTomato, PDPN and CDH5 on whole mount ear skin from *Pax3*$^{Cre/+}$*;Rosa26*^{tdtomato} (**j**, **j'**) or *Myf5*$^{Cre/+}$*;Rosa26*^{tdtomato} (**k**, **k'**) mice at post-natal day 21. Scale bars: a-c''', e', g-g' – 50 µm; d-d', g-g', i-i', j-k' – 100 µm; e – 1 mm; f-f' – 200 µm; h – 500 µm.

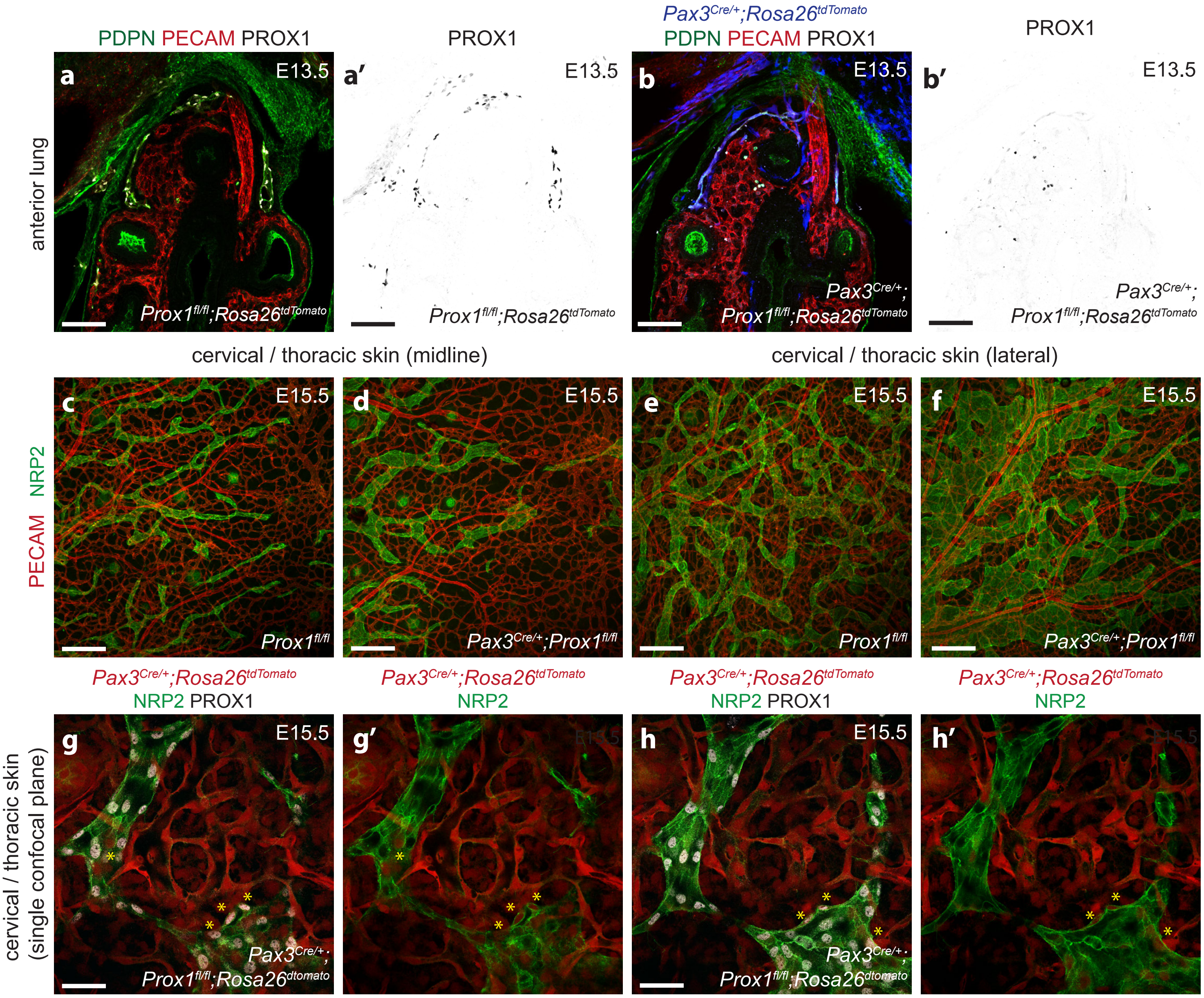

Pax3Cre/+;Rosa26tdTomato
PDPN PECAM PROX1
PROX1
a
a′
b
b′
E13.5
anterior lung
Prox1fl/fl;Rosa26tdTomato
Pax3Cre/+;
Prox1fl/fl;Rosa26tdTomato
cervical / thoracic skin (midline)
cervical / thoracic skin (lateral)
c
d
e
f
E15.5
PECAM NRP2
Prox1fl/fl
Pax3Cre/+;Prox1fl/fl
Pax3Cre/+;Rosa26tdTomato
NRP2 PROX1
NRP2
g
g′
h
h′
cervical / thoracic skin
(single confocal plane)
Pax3Cre/+;
Prox1fl/fl;Rosa26dtomato

Figure S4: Related to Figure 4. Analysis of *Prox1* deletion in the paraxial mesoderm derived cells.

Immunofluorescence for tdTomato, PDPN, PECAM and PROX1 on sagittal vibratome sections at the level of the cardiac outflow tract from $Prox1^{fl/fl};Rosa26^{tdTomato}$ (**a**-**a’**) and $Pax3^{Cre/+};Prox1^{fl/fl};Rosa26^{tdTomato}$ embryos (**b**-**b’**). Immunofluorescence for PECAM and NRP2 on whole mount cervical/thoracic skin from $Prox1^{fl/fl}$ and $Pax3^{Cre/+};Prox1^{fl/fl}$ embryos, at midline (**c**-**d**) and lateral positions (**e**-**f**). Immunofluorescence for tdTomato, NRP2 and PROX1 on whole mount cervical/thoracic skin from $Pax3^{Cre/+};Prox1^{fl/fl}$ embryos (**g**-**h’**) - yellow asterisks highlight $PROX1^+$ cells that are labeled by $Pax3^{Cre/+}; Rosa26^{tdtomato}$ in the cervical/thoracic skin. Scale bars: a, b, d, e, l, m– 50 µm; c, l-m – 100 µm; h-k – 200 µm.
